# Glycemic variability of glycated hemoglobin in patients with type 2 diabetes mellitus and the risk of cardiovascular diseases: a latest systematic review and meta-analysis

**DOI:** 10.3389/fendo.2025.1698360

**Published:** 2025-11-06

**Authors:** Chan Wu, Aijing Li, Qingyi Zhu, Jingyi Guo, Yincheng Li, Xin Gu, Anning Sun, Maoying Wei, Yanbing Gong

**Affiliations:** 1 Dongzhimen Hospital, Beijing University of Chinese Medicine, Beijing, China; 2 Beijing University of Chinese Medicine, Beijing, China

**Keywords:** HbA1c variability, type 2 diabetes mellitus, cardiovascular disease, meta-analysis, diabetes complications

## Abstract

**Objective:**

Glycated hemoglobin (HbA1c) variability is a crucial indicator for evaluating the stability of long-term glycemic control in patients with diabetes mellitus. This study aimed to clarify the association between HbA1c variability and the risk of incident cardiovascular disease (CVD) and mortality in patients with type 2 diabetes mellitus (T2DM) through a systematic review, thereby providing evidence-based support for the early prevention of adverse cardiovascular events in T2DM patients.

**Methods:**

We systematically searched the PubMed, Web of Science, The Cochrane Library, and Embase databases for studies on the association between HbA1c variability and cardiovascular outcomes in patients with T2DM, published from the establishment of each database up to August 5, 2025. Cardiovascular outcomes included the incidence of CVD and CVD-related mortality. Two researchers independently conducted literature screening, data extraction, and risk of bias assessment. Meta-analysis was performed using Review Manager 5.3 software, with hazard ratio (HR) or odds ratio (OR) as the effect size.

**Results:**

A total of 31 cohort studies were included, covering 545,956 participants from 13 countries and regions. The results of the meta-analysis showed that a higher coefficient of variation (CV) of HbA1c was significantly associated with an increased risk of cardiovascular events (HR = 1.32, 95% CI: 1.18–1.49, P < 0.00001; OR = 1.39, 95% CI: 1.22–1.57, P < 0.00001), and also significantly elevated the risk of mortality (HR = 1.35, 95% CI: 1.16–1.57, P < 0.00001). The standard deviation (SD) of HbA1c was also significantly correlated with a higher risk of cardiovascular events (HR = 1.27, 95% CI: 1.17–1.38, P < 0.00001; OR = 1.30, 95% CI: 1.07–1.57, P = 0.008) and a significant increase in mortality risk (HR = 1.27, 95% CI: 1.17–1.37, P<0.00001). The hemoglobin glycation index (HGI) was significantly associated with the risk of cardiovascular events in terms of HR (HR = 1.36, 95% CI: 1.14–1.62, P = 0.0006), but no statistical significance was observed in terms of OR (OR = 1.47, 95% CI: 0.98–2.20, P = 0.06). In contrast, the HbA1c variability score (HVS) showed no significant association with either the risk of cardiovascular events (HR = 1.31, 95% CI: 0.97–1.78, P = 0.08) or mortality risk (HR = 1.00, 95% CI: 0.76–1.31, P = 1.00).

**Conclusions:**

HbA1c variability is positively associated with the risk of adverse cardiovascular events in patients with T2DM. Among the indicators of HbA1c variability, the coefficient of variation (CV), standard deviation (SD), and hemoglobin glycation index (HGI) can serve as significant predictors for the risk of cardiovascular disease (CVD) occurrence and mortality. However, the HbA1c variability score (HVS) did not show significant predictive value in this study.

**Systematic Review Registration:**

https://www.crd.york.ac.uk/prospero/, identifier CRD420251132972.

## 1 Introduction

The global prevalence of diabetes is increasing. Statistics show that there are currently 537 million adults living with diabetes worldwide, with the majority residing in low- and middle-income countries (1). As the cost of disease treatment rises, the burden on healthcare systems will further escalate—this is particularly true when patients in the middle and late stages of diabetes develop diabetes-related complications (2). Cardiovascular disease (CVD) remains the leading cause of mortality and disability among patients with type 2 diabetes mellitus (T2DM), posing a significant public health challenge and imposing a heavy economic burden on countries across all levels of socioeconomic development (3).

There is a complex pathophysiological link between diabetes and cardiovascular complications, and this mechanism has driven extensive research focused on the development of risk prediction models and the optimization of early intervention strategies (4). Although a causal relationship between glycemic control and vascular complications has been clearly established, traditional biochemical markers such as glycated hemoglobin (HbA1c)—which serves as an indicator of peripheral insulin resistance and reflects the average blood glucose concentration over the past 2 to 3 months—are widely used biomarkers for diabetes monitoring and prognosis in clinical practice. However, HbA1c is prone to errors due to factors such as pregnancy and anemia; studies have reported that HbA1c levels are only associated with chronic hyperglycemia (5). In contrast, HbA1c is not correlated with glycemic variability parameters, and thus has certain limitations in reflecting the dynamic characteristics of blood glucose fluctuations and their impact on diabetes-related vascular outcomes.

Recent studies have shown that glycemic variability, especially indicators of HbA1c variability such as standard deviation (SD), coefficient of variation (CV), HbA1c variability score (HVS), and hemoglobin glycation index (HGI), can independently predict cardiovascular-related outcomes and serve as additional predictive indicators for diabetes complications (6). Among these, the standard deviation (SD) and coefficient of variation (CV) of HbA1c are the most commonly used measurement indicators. HVS can more comprehensively reflect the pathophysiological process of vascular complications through multiple mechanisms, including blood glucose dynamic fluctuations induced by it, hypoglycemia-related oxidative stress responses, and persistent chronic inflammatory states (7–9). Therefore, high HVS is closely associated with an increased risk of cardiovascular diseases in patients with diabetes. In addition, for critical adverse events in cardiovascular diseases—such as blood glucose fluctuations caused by acute stress responses during myocardial infarction—the prognostic value of HbA1c decreases significantly (10). In such cases, the hemoglobin glycation index (HGI), by quantifying the difference between measured HbA1c and predicted HbA1c, can more comprehensively assess blood glucose status and timely reflect individual blood glucose fluctuation (11). Although the SD and CV are widely used to assess HbA1c variability, the evidence for HVS and HGI remains fragmentary and inconsistent (12–14). For example, two cohort studies in East Asia reported opposite findings for HVS (15). A Korean study reported that higher HVS was significantly associated with increased Major Adverse Cardiovascular Events (MACE) risk, whereas a contemporary Scottish cohort found no significant link between HVS and CVD events. Similarly, the association between HGI and CVD differs between Western and Korean populations (16, 17). Large, multicenter cohorts in Europe and North America consistently show a linear, positive correlation between HGI and MACE. In contrast, Korean data reveal a U-shaped curve, indicating that both extremely high and extremely low HGI levels are associated with elevated MACE risk. Another source of inconsistency in existing studies lies in the variation of statistical measures used to quantify the association between HbA1c variability indicators and CVD outcomes. Some studies used relative risk (RR) as the measure of association (18, 19), which does not account for the time factor. In contrast, the hazard ratio (HR) incorporates time information and can handle censored data, making it more accurate for evaluating the long-term impact of HbA1c variability on cardiovascular outcomes. In addition, multicenter cohorts published after 2023 have not yet been included in any published synthesis. Therefore, an updated synthesis comparing the consistency and robustness of different indicators is urgently needed.

The relationship between HbA1c variability indicators and the risk of cardiovascular events in patients with T2DM remains not fully understood, especially in diverse populations with different diabetes durations and treatment regimens, which requires more in-depth research (20). Although the variability of glycated hemoglobin has potential clinical significance, current diabetes management guidelines mainly focus on average glycemic control rather than glycemic variability. This study systematically conducts literature search, quality assessment, and quantitative synthesize of prospective/retrospective cohort studies published up to August 2025. Using a random-effects meta-analysis approach, it aims to clarify the independent predictive value of four HbA1c variability indicators (SD, CV, HVS, HGI) for incident cardiovascular events and cardiovascular mortality in patients with type 2 diabetes mellitus.

## 2 Research design and methods

### 2.1 Protocol and registration

This study protocol was prospectively registered in the International Prospective Register of Systematic Reviews (PROSPERO; registration number: CRD420251132972) in advance. This meta-analysis was conducted in strict accordance with the Preferred Reporting Items for Systematic Reviews and Meta-Analyses (PRISMA) statement guidelines (21). Since the included studies were cohort studies (observational studies), the Meta-Analysis of Observational Studies in Epidemiology (MOOSE) guidelines (22) were also followed.

### 2.2 Search strategy

A comprehensive search was conducted using PubMed, Embase, Web of Science, and the Cochrane Library, with no language restrictions applied. The search covered the period from the inception of each database up to August 5, 2025. For the search strategy, we combined Medical Subject Headings (MeSH) terms (23) with text words related to HbA1c variability and cardiovascular disease progression, integrating both controlled terms and free-text terms. The search terms included: ① Glycated Hemoglobin, Glycated Hemoglobin A1c, HbA1c, HbA(1c) variability, HbA(1c) variation; ②Disease, Cardiovascular, Cardiac Events, Adverse Cardiac Event, Major Adverse Cardiac Events, Cardiovascular Diseases; ③ Diabetes Mellitus, Diabetes Insipidus, Diet, Diabetic, Prediabetic State, Scleredema Adultorum, Glucose Intolerance, Gastroparesis, Glycation End Products. Two reviewers (C.W. and A.J.L.) independently screened all titles and abstracts, and selected full texts of potentially relevant articles. Disagreements were resolved through debate, discussion, or consultation with a third investigator (Q.Y.Z.). Meanwhile, EndNote X20 was used for literature analysis and management.

### 2.3 Selection of studies (PICOS)

P: Inclusion Criteria:

1. Studies investigating HbA1c variability indicators (including SD, CV, HVS, and HGI).
2. Adult patients (aged ≥18 years) with a confirmed diagnosis of type 2 diabetes.
3. Studies that included patients without a history of cardiovascular-related events at baseline. To avoid reverse causation, where acute events drive HbA1c fluctuations through stress-induced hyperglycemia, we restricted the analysis to a primary prevention population, ensuring that any observed association reflects the prospective direction of interest.
4. Studies from which hazard ratios (HRs), relative risks (RRs), or odds ratios (ORs) and their corresponding 95% confidence intervals (CIs) could be extracted.

Full texts of potentially relevant studies were downloaded and reviewed for inclusion.

Exclusion Criteria:

1. Studies involving patients with type 1 diabetes, gestational diabetes, a history of major cardiovascular events (e.g., myocardial infarction, stroke) at baseline, an expected lifespan shorter than the follow-up period, or an insufficient number of HbA1c measurements during the follow-up period.
2. Reviews, case reports, practice guidelines, commentaries, *in vitro* or animal studies, *post-hoc* analyses of randomized controlled trials, or analyses unrelated to the topic of this study.
3. Non-English articles. Following the practice of previous systematic reviews (24, 25), this study extracted data only from full-text articles published in English to ensure consistency in data extraction and interpretation. To assess the potential impact of this limitation, we sensitivity-checked the abstracts of non-English studies and found no directional change, which indicates the exclusion of these articles is unlikely to alter our conclusions.
4. Duplicate articles; if identical literature was identified, only one article was included.
5. Articles for which the full text was unavailable, relevant valid data could not be extracted, or there were obvious errors in the data.

I: High levels of glycated hemoglobin (HbA1c) variability: Standard Deviation (SD) and adjusted Standard Deviation (Adj-SD); Coefficient of Variation (CV = SD/Mean); HVS: HbA1c variability score; HGI: hemoglobin glycation index.

C: The control group consisted of a patient population with low HbA1c variability. Studies typically compared the risk differences between the highest quartile group and the lowest quartile group. Comparison condition: Logistic or Cox regression analysis was used for outcome risk prediction.

O: Cardiovascular events:

Primary outcomes: CVD (events of myocardial infarction, ischemic heart disease, heart failure, nonfatal ischemic stroke, or peripheral vascular disease).

Secondary outcomes: Cardiovascular mortality.

S: Prospective Cohort Study or Retrospective Cohort Study.

### 2.4 Quality assessment

The risk of bias was also independently assessed by C.W. and A.J.L. For cohort studies and *post-hoc* analyses, in accordance with the recommendations of the Cochrane Collaboration, the Newcastle-Ottawa Scale (NOS) (26) was selected to evaluate study quality, with details available at http://www.ohri.ca/programs/clinical_epidemiology/oxford.asp. In this context, a 9-star rating system (maximum score of 9 stars) was used, which is divided into three domains: selection of participants (0–4 stars), comparability of study groups (0–2 stars), and determination of outcomes (0–3 stars). Studies with a score of ≥ 8 stars were classified as low risk of bias, those with 6–7 stars as moderate risk, and those with 5 stars as high risk.

### 2.5 Data analysis and synthesis

The meta-analysis was conducted using Review Manager (RevMan) Version 5.3. Stratified analyses were performed based on the variations in data regarding HbA1c variability indicators and types of effect sizes among the included studies, with subgroup analysis results and pooled values presented separately. Given the methodological differences between HR and OR, independent analyses were conducted for each. A random-effects model was used for data pooling.

The results were visualized as a forest plot using the inverse variance method. Data were entered into RevMan 5.3 in the form of the natural logarithm of risk estimates (HR or OR) and their standard errors. When necessary, the standard error was derived from the confidence interval (CI) using the formula: (ln upper CI - ln lower CI)/(2×1.96). A random-effects model was used to calculate the I² statistic for assessing heterogeneity, with the following judgment criteria: 0%-25% indicates very low heterogeneity, 25%-50% indicates low heterogeneity, 50%-75% indicates moderate heterogeneity, and >75% indicates high heterogeneity (27). Subgroup analyses were performed based on dimensions such as HbA1c variability indicators, sample size, region, study design, follow-up duration of HbA1c variability, and comparison levels of HbA1c variability to identify the sources of heterogeneity. Sensitivity analyses were conducted to evaluate the robustness of the results by excluding low-quality studies, removing studies that only reported relative risk (RR), excluding studies with short or unclear average follow-up duration, and re-analyzing using a fixed-effects model instead. Publication bias was assessed using Egger’s test and funnel plots. If publication bias existed, the trim-and-fill method was used to estimate the impact of missing studies. A P-value < 0.05 was set as the threshold for statistical significance in all analyses. Subgroup analyses, Egger’s test, Trim-and-Fill adjustment results, and sensitivity analyses have been provided in **Appendix A** (**Supplementary Tables S1–S6**) to ensure transparency and reprehensibility of the findings.

### 2.6 Clinical definitions

SD was calculated as $\sqrt{\Sigma_{k=1}^{n}\frac{{\left(x_{i}-\bar{x}\right)}^{2}}{n-1}}$ and adjusted SD was calculated as $SD/\sqrt{\frac{n}{n-1}}\cdot CV$ was calculated as $SD/\bar{X}$ and adjusted CV was calculated as $CV/\sqrt{\frac{n}{n-1}}$, where n = total number of HbA1c measurements, $X_{i}=$ serially measured HbA1c, and $\bar{X}=\;$ mean of HbA1c (27). HVS was the number of HbA1c changes >0.5% over the total number of HbA1c measurements (17). HGI was calculated as measured HbA1c minus predicted HbA1c from fasting blood glucose (FBG) levels (28).

The diagnostic criteria for T2DM were as follows: (1) FBG ≥7.0 mmol/L; (2) 2-h oral glucose tolerance test or casual plasma glucose level ≥11.1 mmol/L; (3) HbA1c ≥ 6.5%; or (4) prior diagnosis of T2DM.

## 3 Results

### 3.1 Characteristics of included studies

**Figure 1:**
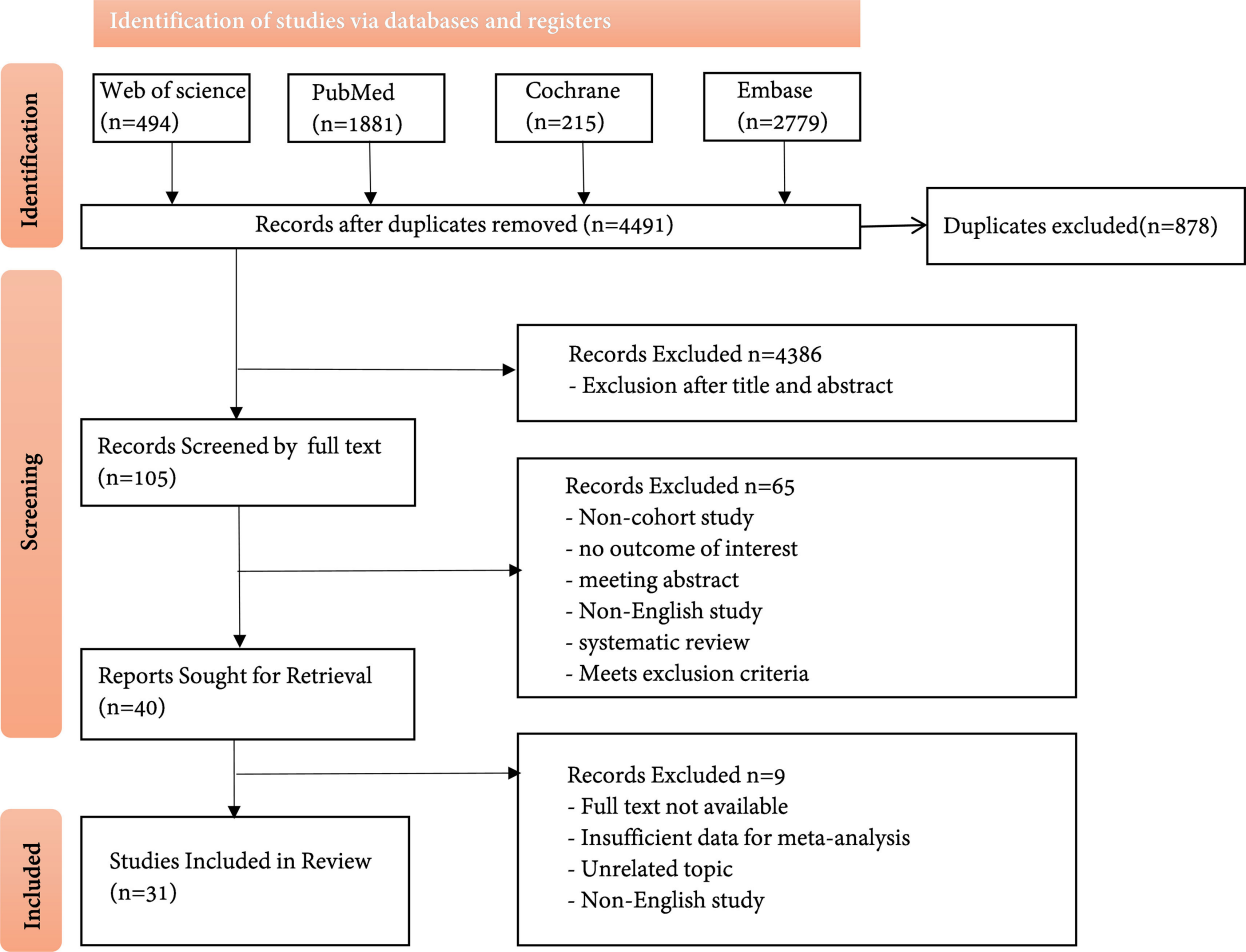
PRISMA flow diagram outlining the selection process that was undertaken for the systematic review and meta-analysis.

Through the search method described above, a total of 5,369 articles were retrieved. After removing 891 duplicate articles, 4,386 articles that did not match the research topic were excluded after a preliminary review of titles and abstracts, resulting in 105 articles after initial screening. Subsequently, 65 articles were excluded after a detailed full-text review, including non-cohort studies, studies involving non-type 2 diabetes patients, studies without relevant results, conference abstracts, non-English studies, and systematic reviews, leaving 40 articles. Finally, articles that could not be downloaded and had incomplete data were excluded, resulting in 31 articles. The search process is shown in **Figure 1**.

All 31 included articles were cohort studies, covering 545,956 participants from 13 countries and regions. The basic characteristics of the included articles are shown in **Table 1**. Six studies (29, 38, 45–47, 49) reported adjusted HRs or ORs for high SD versus low SD, involving a total of 140,260 participants, with the sample size ranging from 689 to 101,533. These studies were conducted in multiple countries including China, the United States, Brazil, Japan, and Thailand, with an average follow-up period ranging from 3 years to 15.9 years. Among the included studies, 20 reported (19, 29–33, 35–38, 41, 42, 45–49, 52–54) SD values, and 3 (30, 32, 47) provided adjusted SD values. The average glycated hemoglobin (HbA1c) level ranged from 6.8% to 8.69%. Sixteen studies (19, 29, 30, 32, 33, 35–37, 41, 42, 45, 47, 49, 52–54) explored the relationship between SD and the incidence of diabetes-related adverse cardiovascular events, while seven studies (31, 36, 38, 41, 46, 48, 54) investigated the relationship between SD and mortality from diabetes-related cardiovascular events; details can be found in **Table 1**.

**Table 1:** Characteristics of the studies considered in the meta-analysis.

Study(Author, year)	Design(Type of study)	Number(male%)	Age at enrolment	Area (follow-up time, years)	Inclusion criteria	HbA1c variability and follow-up time	Mean HbA1c(%)	Outcome	Variable adjustment	NOS score
Bouchi R et al. (2012) (29)	observational cohort study	689(57.18%)	65±11	Japan(3.3(1.0-6.3))	patients with T2DM without CVD	SD:0.6±0.42, CV:8.0±4.6, ≥12months	7.8±1.2	Incidence of CVD	Adjusted for age, sex, duration of diabetes, the presence of proliferative diabetic retinopathy, smoking status, the use of insulin, renin–angiotensin system inhibitors, antiplatelet agents, and statins, hemoglobin, uric acid, eGFR, urinary ACR at baseline, BMI, SBP and DBP, triglycerides, HDL-C and LDL-C levels	7
Luk AO et al. (2013) (30)	prospective cohort study	8439(47.0%)	57.6±13.2	Hong Kong, CHINA(median follow-up period of 7.2 years)	patients with T2DM	Adjusted SD:NA, median number of HbA1c measurements was 10(IQR: 5–17)	NA	CVD (events of myocardial infarction , ischemic heart disease, heart failure, nonfatal ischemic stroke or peripheral vascular disease)	Adjusted for age, gender, smoking history, diabetes duration, BMI, waist circumference, SBP/DBP, LDL-C, HDL-C, log triglyceride, log urine ACR, eGFR, haemoglobin and baseline medication use including the use of ACE inhibitors/ARB, antihypertensive drugs, lipid-lowering drugs, oral hypoglycaemic drugs and insulin.	7
Takao T et al. (2014) (31)	observational cohort study	754(81.83%)	54.4±10.0	Japan(15.9(10.3-16.6))	patients with T2DM	SD:NA, CV:NA, ≥24months	8.0±1.7	Mortality of CVD	Adjusted for age, sex, mean HbA1c, the number of HbA1c measurements, duration of diabetes, mean BMI, mean SBP, mean TC/HDL-C, and current smoker.	8
Yang HK et al. (2015) (32)	observational cohort study	595(58.32%)	64.88	Korea(9.63±9.04)	patients with T2DM without history of CVD	Adjusted-SD: 0.48(0.45, 0.51), CV:6.98 (6.64, 7.33), 973 days	8.69±2.30	Incidence of CACS	Adjusted for age, sex, duration of diabetes, HbA1c-MEAN, HbA1c-SD groups, total cholesterol level, use of insulin and statin.	9
Bonke FC et al. (2016) (33)	observational cohort study	13777(54.70%)	67.4±11.1	Bavaria(9.0±6.7)	patients with T2DM without CVD	SD:NA, ≥5years	8.2±1.4	Incidence of CVD	Adjusted for age, sex, smoking status, absolute HbA1c value at baseline, diabetes history of more than 8 years (i.e. the median duration at baseline), peripheral artery disease, the presence of diabetic complications (retinopathy, neuropathy or nephropathy) and a record of previous myocardial infarction, stroke or diabetes-related emergency admission	8
Ahn CH et al. (2017) (34)	prospective cohort study	1248(59.54%)	55.3±11.3	Korea(1.07(1.05-1.09))	patients with T2DM	HGI:0.05± 0.98, NA	7.0±1.7	Incidence of CVD	Adjusted for age, sex,BMI, smoking, hypertension, dyslipidemia, family history of CVD, HDL-C, LDL-C, and hsCRP level.	8
Lee MY et al. (2017) (35)	longitudinal cohort study	8259(52%)	62.0±11.9	Taiwan, China(6.3±1.3)	patients with T2DM	SD:0.84±0.58, mean follow-up of 6.3 years	7.5±1.2	Incidence of CVD	Adjusted for age, gender, a history of hypertension, retinopathy and neuropathy, SD of HbA1C, mean HbA1C, triglyceride, HDL-cholesterol, eGFR, and medications use, including ACEI and/or ARB, aspirin, statin and/or fibrate, and insulin.	7
Cardoso CRL et al. (2018) (36)	prospective cohort study	654(38.1%)	60.1(9.6)	Rio de Janeiro, Brazil(9.3(5.2-10.8))	patients with T2DM	SD:NA, CV:NA, NA	8.1(1.9)	Incidence and mortality of CVD	Adjusted for age, sex number of HbA1c or FG measurements, diabetes duration, BMI, smoking status, physical inactivity, arterial hypertension, number of anti-hypertensive drugs in use, ambulatory 24-h SBP, presence of micro- and macrovascular complications at baseline, serum mean HDL-C and LDL-C, use of insulin, statins and aspirin, mean fasting glycemia and HbA1c.	8
Gu J et al. (2018) (37)	retrospective cohort study	201(NA)	65.2±7.5	China(7.3±0.5)	patients with T2DM	SD:NA, CV:NA, median follow-up of 7.3 years	7.1±0.5	Incidence of CVD	Adjusted for age, gender, SBP, DBP, HbA1c-mean, HbA1c-SD, eGFR, BMI, duration of T2DM and hypertension, atrial fibrillation, medical treatment, LAD, LVMI, E/E′, and LVEF.	7
Kim MK et al. (2018) (17)	prospective cohort study	1302(41.94%)	55.5±10.9	Korea(6.5±6.6)	patients with T2DM	HGI:−0.01 ± 1.34, median follow-up of 11.1 years	8.4 ± 1.8	Incidence and mortality of CVD	Adjusted for age, sex, duration of diabetes, presence of hypertension, BMI, eGFR, albuminuria, smoking, insulin treatment, and use of sulfonylurea and aspirin.	8
Kaze AD et al. (2020) (38)	prospective cohort study	3560(37.9%)	58.4(6.7)	U.S.A.(median follow-up period of 6.8 years (IQR 6.0-7.4))	patients with T2DM	SD:NA, ≥36months	7.0(1.0)	Mortality of CVD	Adjusted for age, sex, race/ethnicity, randomization arm, BMI, current smoking, alcohol drinking, use of antihypertensive medications, TC/HDL-C, eGFR, duration of diabetes, average SBP and average HbA1c.	8
Li S et al. (2020)-1 (39)	retrospective cohort study	21352(54.6%)	63.3±11.1	Scotland(6.8(4.6-11.2))	patients with T2DM	HVS:NA, NA	7.7±2.0	Incidence of CVD	Adjusted for sex, index age, calendar year, Scottish Index of Multiple Deprivation quintiles, ever smoking, hypertension at baseline, BMI at baseline, HDL-C at baseline, eGFR at baseline, antiplatelet therapy at baseline, and CCI (≥1 vs. 0).	6
Li S et al. (2020)-2 (40)	prospective cohort study	396(50.50%)	46.30±10.9	China(2.3(1.8-3.1))	patients with T2DM	CV:16.73±7.87, NA	6.7±1.4	Incidence of CVD(coronary artery plaque progression)	Adjusted for age, sex, BMI, diabetes duration, smoking, alcohol consumption, hypertension, hypoglycemia rate, triglyceride, LDL-C, HDL-C, FPG, 2h-FBG, HbA1c, eGFR, UA, HOMA-IR, UACR, and medications.	8
Wan EYF et al. (2020) (41)	prospective cohort study	147811(46.0%)	64.2(10.0)	Hong Kong, China(median follow-up period of 7.4 years)	patients with T2DM without CVD	SD:0.8(0.7), ≥12months	7.5(0.9)	Incidence and mortality of CVD	Adjusted for gender, age, smoking status, duration of diabetes, BMI, SBP and DBP, LDL-C, eGFR, Charlson's comorbidity index, and use of anti-hypertensive drugs, oral antidiabetic drugs, including metformin, sulphonylureas and other oral diabetes drugs, insulin, and lipid-lowering agents.	9
Akselrod D et al. (2021) (42)	retrospective cohort study	2866(43.3%)	58.6	southern region of Israel(NA)	patients with T2DM and having a normal kidney function	SD:1.2, 11 years	7.8	Incidence of IHD	Adjusted for insulin use, gender, age, ischemic heart disease, BMI ≥ 30, smoking.	7
Moosaie F et al. (2021) (43)	cohort study	1500(54.8%)	62.12(8.66) 61.21(9.18) 62.58(11.06) 58.16(10.06)	Iran(median follow‐up period of 10 years)	patients with T2DM	CV:0.165±0.012, median follow‐up of 10 years	7.834(0.932) 7.862(1.102) 7.903(1.291) 7.817(1.546)	Incidence of CVD	Adjusted for age, gender, duration of diabetes, lipid lowering drugs, antidiabetic drugs.	7
Sato M et al. (2021) (44)	prospective cohort study	4532(47.51%)	65.1±9.5 64.5±10.0 63.5±10.0 61.9± 0.9 60.2±11.5	Japan(median follow‐up period of 3.17 years)	patients with T2DM	CV:Q1:0.59–3.95, Q2:3.95–5.56, Q3:5.56–7.32, Q4:7.32–10.07, Q5:10.07–45.42, median follow‐up of 38 months	7.0±0.8 7.3±0.9 7.5±0.9 7.7±1.1 8.1±1.2	Incidence of CVD	Adjusted for sex, age, smoking habits, duration of diabetes, BMI, group of statin therapy, hypertension, diabetic nephropathy, diabetic neuropathy, eGFR at baseline, the number of HbA1c measurements and mean‐HbA1c.	8
Shen Y et al. (2021) (45)	retrospective cohort study	29,260(45.9%)	66.0±11.6	Louisiana, U.S.A.(mean follow-up period of 4.18 years)	patients with T2DM	CV:NA, SD:NA, NA	6.3±0.7 6.7±0.8 7.5±1.1 8.6±1.5	Incidence of CVD	Adjusted for age, race, sex, smoking, BMI, SBP, non-HDL/HDL ratio, eGFR, insurance type, hypoglycaemic events, glucose-lowering medications, antihypertensive medications, lipid-lowering medications, and antiplatelet and anticoagulant medications by category differences, as well as the updated mean value of HbA1c.	7
Ceriello A et al. (2022) (46)	retrospective cohort study	101,533(55.63%)	64(52-72) 68.0(61.0–74.0) 66.0(58.0–72.0) 63.0(55.0–71.0) 60.0(52.0–68.0)	Swedish(mean follow-up period of 4.4 years)	patients with T2DM	SD:NA, NA	NA	Mortality of CVD	Adjusted for age, gender, duration of diabetes, body weight, smoking, values of HbA1c, SBP/DBP, TC, HDL, LDL, triglycerides, albuminuria, eGFR, retinopathy, treatment for diabetes, hypertension, dyslipidemia, and aspirin.	8
Ma C et al. (2022) (47)	prospective cohort study	2161(38.45%)	NA	China(NA)	patients with T2DM	SD:NA, CV:NA, NA	NA	Cardiovascular events	Adjusted for gender, age, duration of type 2 diabetes, BMI, smoking, baseline concomitant disease, triglycerides, LDL-C, blood pressure, anti-hyperglycemic therapy, and ACEI or ARB treatment,average HbA1c.	7
Wu TE et al. (2022) (48)	prospective cohort study	1869(50.4%)	63.2±12.7	Taiwan, China(median follow-up period of 9.5 years)	patients with T2DM	SD:0.728±0.528%, 5 years(10-42)	8.0±1.77	Mortality due to CVD	Adjusted for HbA1c-mean or HbA1c-SD, age, sex, diabetes duration, blood pressure, BMI, TC, HDL-C, triglyceride, and smoking status.	7
Hsu JC et al. (2023) (8)	retrospective cohort study	45,436(48.36%)	>50	Taiwan, China(median follow‐up period of 5.38 years)	patients with T2DM	HVS:NA, NA	6.34(0.74) 6.87(0.99) 7.48(1.38) 8.05(1.78)	Mortality of CVD	Adjusted for age, sex, baseline BMI, hypertension, coronary artery disease, average of fasting glucose, average HbA1c, baseline eGFR, model 2 plus medications (metformin, SGLT2i, DDP4i , GLP‐1 agonist).	7
Kim H et al. (2023) (15)	retrospective cohrot study	4817(51.5%)	59.9±9.8	Korea(9)	patients with T2DM	HVS:31.2 ± 22.8, NA	7.2±1.4	Incidence of CVD	Adjusted by age, sex, BMI.	7
Manosroi W et al. (2023) (49)	prospective cohort study	3057(45.67%)	64.7±9.2	Thailand(median follow‐up period of 4.51 years)	patients with prediabetes and T2DM	SD:NA, NA	7.3±1.5	Incidence of CVD(MACE)	Adjusted for age, educational level, sex, BMI, established atherosclerotic cardiovascular disease status, SBP, smoking status, mean glycated hemoglobin, lipid profiles, creatinine level number of glycated hemoglobin measurements,plus antihypertensive medications, diabetes medications, lipid‐lowering agents, and antiplatelet and/or anti‐coagulant.	8
Zhang F et al. (2023) (50)	retrospective cohort study	820(51.7%)	56.9±14.6	China(median follow‐up period of 3.67 (1-16.5) [IQR: 2.25,5.83] years)	peritoneal dialysis patients with T2DM and over 18 years old	HVS:NA, NA	7.0±2.3	Incidence of MACE	Adjusted for time-weighted average ­HbA1c, age, sex, cardiovascular disease history, BMI, hemoglobin, albumin and C-reactive protein.	8
Cardoso CRL et al. (2024) (16)	prospective cohort study	687(38.4%)	60.1±9.5	Rio de Janeiro, Brazil(10.6(6.3,13.2))	patients with T2DM	HGI:0±1.6, NA	8.0±1.9	Incidence of CVD	Adjusted for age sex, BMI, physical activity, smoking status, diabetes duration, pre-existent macrovascular and microvascular complications, SBP, serum LDL-C, use of insulin, aspirin and statins, number of antihypertensive drugs in use, HGI and HbA1c parameters.	7
Lin CC et al,(2024) (51)	retrospective cohort study	3628(53.25%)	66.23±10.94	China(≥0.25 years)	patients with T2DM	CV:8.89 ± 7.85, NA	7.70±1.56	Mortality of CVD	Adjusted for baseline sociodemographic factors, lifestyle behavior, diabetes-related variables, HbA1c, complications, medication, and glycemic variation.	6
Liu X et al. (2024) (19)	retrospective cohort study	147(58.86%)	62.07±8.86 63.80±9.95 66.49±12.22	China(NA)	patients with T2DM	SD:NA, CV:NA, NA	NA	Incidence of CVD	Adjusted for sex, age, duration, SBP, DBP, LDL, use of insulin using and HbA1c.	6
Maajani K et al. (2025) (52)	retrospective cohort study	2078(44.8%)	64.7(10.6)	Iran(median follow‐up period of 2.67 years)	patients with T2DM	SD:0.23±0.06, CV:3.5±0.9, NA	6.8±0.82	Incidence of CVD	Adjusted for BMI, SBP and DBP, HbA1c, FBS and TC, HDL-C, LDL-C and Triglyceride, SGL-2, other oral medications, GLP-1, insulin, antihypertensive drugs, lipid-lowering drugs, anti-platelet drugs, age, sex, duration of disease, the baseline and lagged value of time-varying covariates.	7
Teh XR et al. (2025) (53)	retrospective cohort study	40,662(38.3%)	57.2(13.9)	Thailand(10)	patients with T2DM	SD:0.67(0.87), CV:0.07(0.08), 3–6 months	7.7(2.0)	Incidence of CVD	Adjusted for age, gender, insurance scheme, BMI, TC, LDL, HDL, triglyceride, haemoglobin, SBP/DBP, hypertension, dyslipidemia, presence of T2D complications (CVD, DR, or CKD) prior to the outcome of interest, medication use in terms of drug classes (biguanides, sulphonylurea, insulin, alpha- glucosidase inhibitors, DPP-4i, GLP1-RA, TZD, SGLT2i, meglitinides, statins) and the number of antihypertensive drugs.	8

Studies are presented in chronological order of publication.

HbA1c, glycated hemoglobin A1C; T2DM, Type 2 Diabetes Mellitus; NOS, Newcastle-Ottawa Scale; CVD, cardiovascular disease; BMI, mean body mass index; SD, standard deviation; CV, coefficient of variation; eGFR, estimated glomerular filtration rate; ACR, albumin-creatinine ratio; SBP, systolic blood pressure; DBP, diastolic blood pressure; HDL-C, high-density lipoprotein cholesterol; LDL-C, low-density lipoprotein cholesterol; NA, not available; IQR, interquartile range; ACE, angiotensin-converting enzyme; ARB, angiotensin II receptor blocker; TC, total cholesterol; CACS, Coronary Artery Calcium Score; HGI, hemoglobin glycation index; hsCRP, high-sensitivity C-reactive protein; FG, fasting glucose; LAD, left atrium diameter; LVMI, left ventricular mass index; E/E’, E wave to e' wave ratio; LVEF, left ventricular ejection fraction, HVS, HbA1c variability score; CCI, Charlson Comorbidity Index; FPG, fasting plasma glucose; 2h-FBG, 2-hour fasting blood glucose; UA, uric acid; HOMA-IR, Homeostatic Model Assessment of Insulin Resistance; UACR, urine albumin-to-creatinine ratio; IHD, ischemic heart disease; HDL, high-density lipoprotein; LDL, low-density lipoprotein; SGLT2i, sodium-glucose cotrans-porter-2 inhibitors; DPP-4i, dipeptidyl peptidase-4 inhibitors; GLP-1, Glucagon-Like Peptide-1 agonist; MACE, major adverse cardiovascular events; DR, diabetic retinopathy; CKD, chronic kidney disease; GLP1-RA, glucagon-like peptide-1 agonists; TZD, thiazolidinedione.

Four studies (29, 31, 45, 47) reported HRs or ORs for the high versus low coefficient of variation (CV) groups, involving a total of 33,610 participants, with the sample size ranging from 689 to 29,260. These studies were conducted in Mainland China, the United States, Japan, and Iran, with a follow-up period ranging from 3.3 to 10.0 years. Among the included studies, fourteen (19, 29, 31, 32, 36, 37, 40, 43–45, 47, 51, 53, 54) reported CV values, and the average HbA1c level ranged from 6.7% to 8.69%. Twelve studies (19, 29, 32, 36, 37, 40, 43–45, 47, 53, 54) explored the relationship between CV and the incidence of diabetes-related adverse cardiovascular events, while four studies (31, 36, 51, 54) investigated the relationship between CV and mortality from diabetes-related adverse cardiovascular events.

Regarding the HVS, four studies (8, 15, 39, 50) reported adjusted HRs or ORs, involving a total of 72,425 participants, with the sample size ranging from 820 to 45,436. These studies were conducted in Mainland China, Taiwan (China), the Democratic People’s Republic of Korea, and Scotland. The average follow-up period was 3 to 9 years, and the average HbA1c level ranged from 7.0% to 8.05%. Three of the studies (15, 39, 50) explored the relationship between HVS and the incidence of cardiovascular events, while one study (8) investigated the relationship between HVS and the mortality of cardiovascular events. Three studies (16, 17, 34) were included in the meta-analysis of the HGI, involving a total of 3,237 participants, with the studies conducted in South Korea and Brazil. The average HbA1c level ranged from 7.0% to 8.4%.

Meanwhile, the Newcastle-Ottawa Scale (NOS) was used to evaluate the quality of all included cohort studies. Among the 31 articles, 3 scored 6 points (19, 39, 51), 13 scored 7 points (8, 15, 16, 29, 30, 35, 37, 42, 43, 45, 47, 48, 52), 13 scored 8 points (17, 31, 33, 34, 36, 38, 40, 44, 46, 49, 50, 53, 54), and 2 scored 9 points (32, 41); all were articles with low to moderate risk of bias. The detailed NOS scores of the included articles are shown in **Table 1**.

### 3.2 HbA1c variability and incidence of CVD outcomes

#### 3.2.1 HbA1c-SD and the incidence of cardiovascular disease

**Figure 2:**
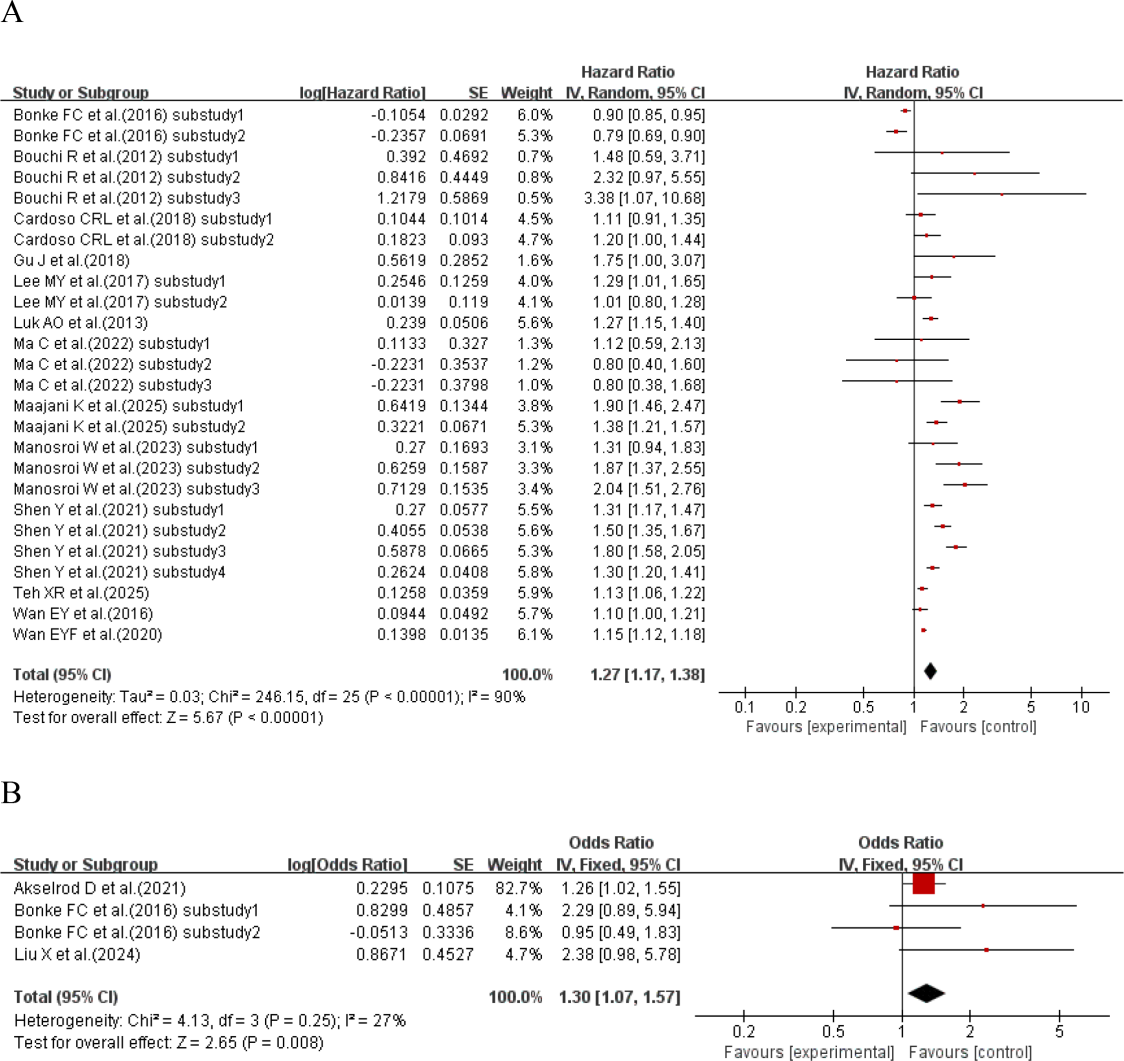
Forest plot showing the association between HbA1c variability (HbA1c-SD) and cardiovascular disease incidence in patients with T2DM, including **(A)** Hazard Ratio (HR) and **(B)** Odds Ratio (OR).

When the effect size was HR, 13 studies (29, 30, 33, 35–37, 41, 45, 47, 49, 52–54) with a total of 26 sub-studies explored the relationship between HbA1c-SD and the incidence of cardiovascular disease. There was heterogeneity among the studies (I²=90%, P<0.00001), so a random-effects model was used for analysis. The meta-analysis results showed that compared with type 2 diabetes patients with lower HbA1c-SD, the incidence of cardiovascular disease in type 2 diabetes patients with higher HbA1c-SD increased by 27% (HR = 1.27, 95%CI 1.17-1.38, P<0.00001). When the effect size was OR, 3 studies (19, 32, 42) with a total of 4 sub-studies explored the relationship between HbA1c-SD and the incidence of cardiovascular disease. There was no heterogeneity among the studies (I²=27%, P = 0.25), so a fixed-effects model was used for analysis. The meta-analysis results showed that higher HbA1c-CV was a risk factor for cardiovascular disease in type 2 diabetes patients (HR = 1.30, 95%CI 1.07-1.57, P = 0.008), as shown in **Figure 2**.

#### 3.2.2 HbA1c-CV and the incidence of cardiovascular disease

**Figure 3:**
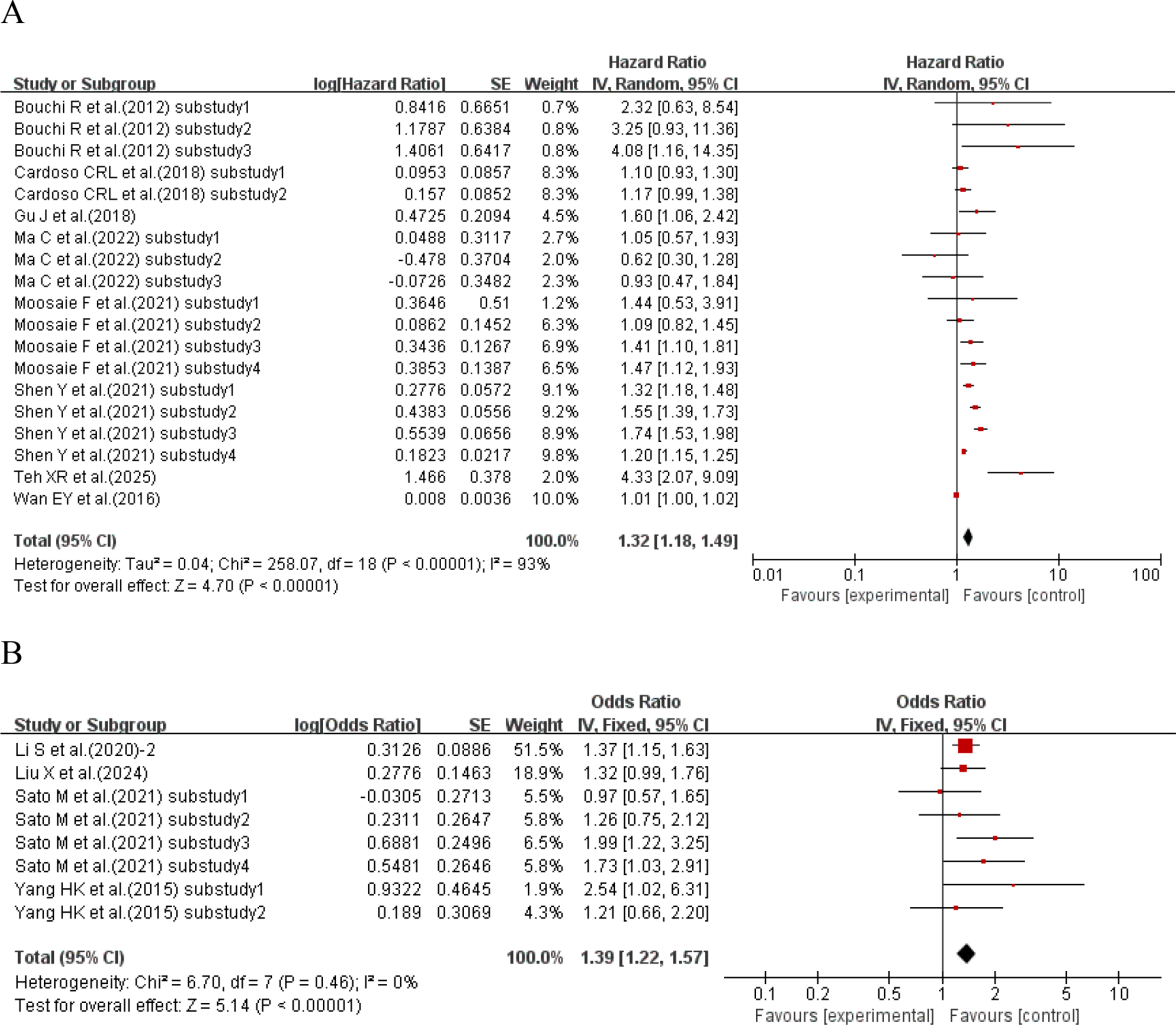
Forest plot showing the association between HbA1c variability (HbA1c-CV) and cardiovascular disease incidence in patients with T2DM, including **(A)** Hazard Ratio (HR) and **(B)** Odds Ratio (OR).

When the effect size was HR, 8 studies (29, 36, 37, 43, 45, 47, 53, 54) with a total of 19 sub-studies explored the relationship between HbA1c-CV and the incidence of cardiovascular disease. There was heterogeneity among the studies (I²=93%, P<0.00001), so a random-effects model was used for analysis. The meta-analysis results showed that compared with type 2 diabetes patients with lower HbA1c-CV, the incidence of cardiovascular disease in type 2 diabetes patients with higher HbA1c-CV increased by 32% (HR = 1.32, 95%CI 1.18-1.49, P<0.00001). When the effect size was OR, 4 studies (19, 32, 40, 44) with a total of 8 sub-studies explored the relationship between HbA1c-CV and the incidence of cardiovascular disease. There was no heterogeneity among the studies (I²=0%, P = 0.46), so a fixed-effects model was used for analysis. The meta-analysis results showed that higher HbA1c-CV was a risk factor for cardiovascular disease in type 2 diabetes patients (OR = 1.39, 95%CI 1.22-1.57, P<0.00001), as shown in **Figure 3**.

#### 3.2.3 HbA1c-HGI and the incidence of cardiovascular disease

**Figure 4:**
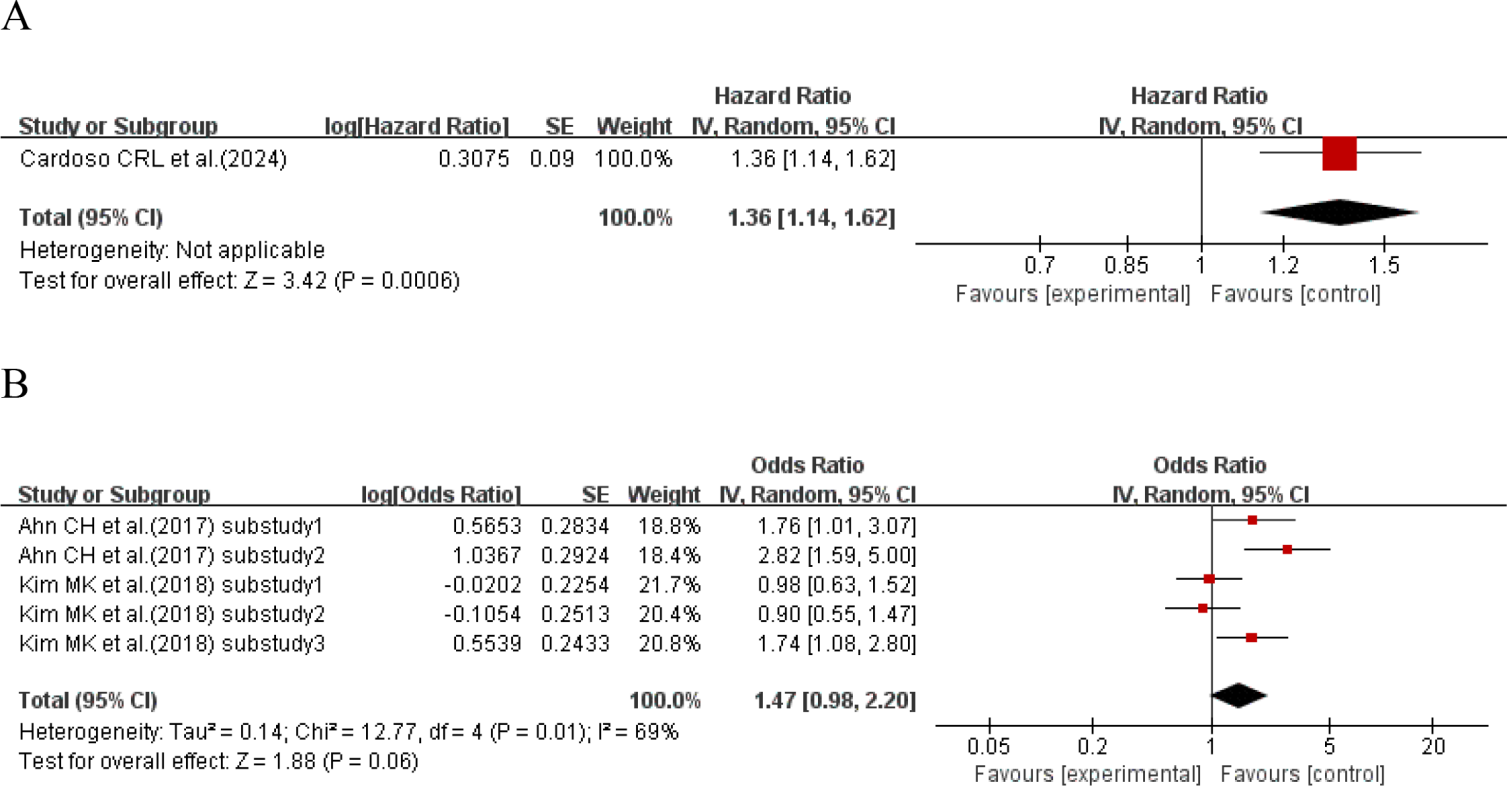
Forest plot showing the association between HbA1c variability (HbA1c-HGI) and cardiovascular disease incidence in patients with T2DM, including **(A)** Hazard Ratio (HR) and **(B)** Odds Ratio (OR).

When the effect size was HR, one study (16) explored the relationship between HbA1c-HGI and the incidence of cardiovascular disease. The results showed that compared with type 2 diabetes patients with lower HbA1c-HGI, the incidence of cardiovascular disease in type 2 diabetes patients with higher HbA1c-HGI increased by 36% (HR = 1.36, 95%CI 1.14-1.62, P = 0.0006). When the effect size was OR, 2 studies (17, 34) with a total of 5 sub-studies explored the relationship between HbA1c-HGI and the incidence of cardiovascular disease. There was heterogeneity among the studies (I²=69%, P = 0.01), so a random-effects model was used for analysis. The results showed a positive trend that higher HbA1c-HGI might increase the risk of cardiovascular disease (P = 0.06 > 0.05), with no statistically significant difference, as shown in **Figure 4**.

#### 3.2.4 HbA1c-HVS and the incidence of cardiovascular disease

**Figure 5:**
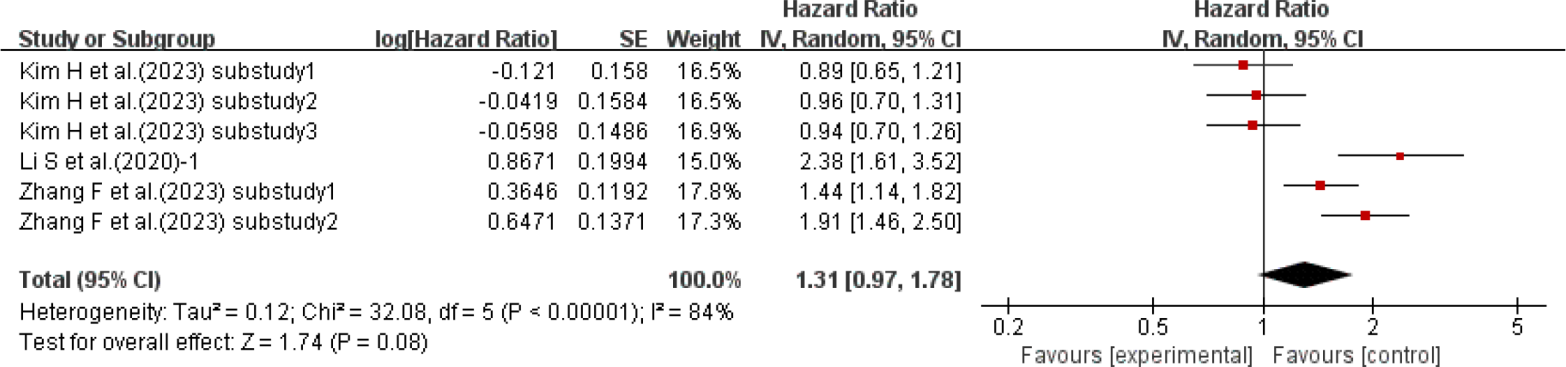
Forest plot of cardiovascular incidence data: HRs for HbA1c-HVS based on published reports of T2DM.

Three studies (15, 39, 50) with a total of 6 sub-studies used HR as the effect size to explore the relationship between HbA1c-HVS and the incidence of cardiovascular disease. There was heterogeneity among the studies (I²=84%, P<0.00001), so a random-effects model was used for analysis. The results showed a positive trend that higher HbA1c-HVS might increase the risk of cardiovascular disease (P = 0.08 > 0.05), but there was no statistically significant difference. Details are shown in **Figure 5**.

### 3.3 HbA1c variability and mortality of CVD outcomes

#### 3.3.1 HbA1c-SD and cardiovascular disease mortality

**Figure 6:**
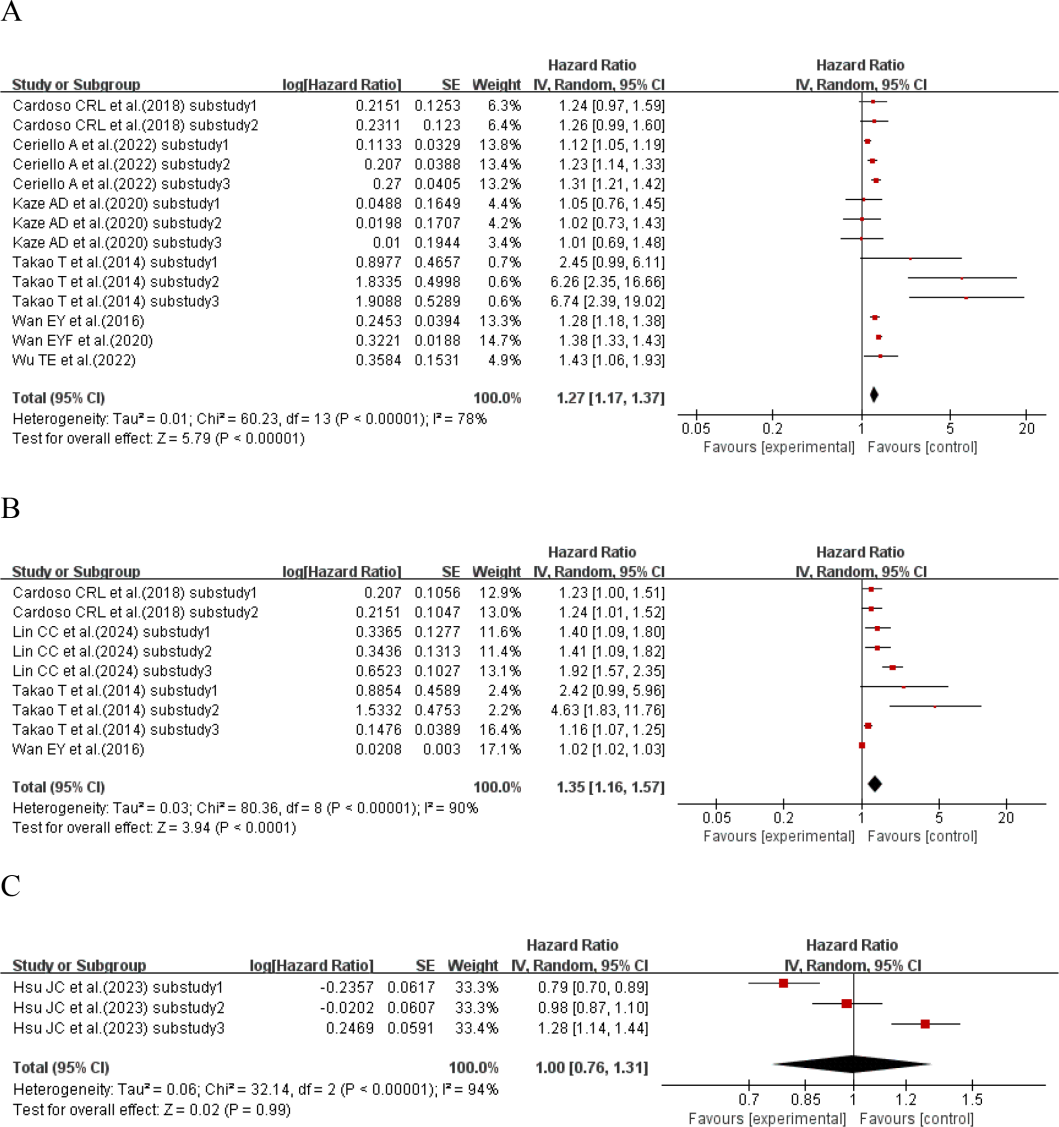
Forest plot of cardiovascular mortality in patients with type 2 diabetes mellitus from published reports, presenting Hazard Ratios (HRs) for **(A)** HbA1c-SD, **(B)** HbA1c-CV, and **(C)** HbA1c-HVS.

Seven studies (31, 36, 38, 41, 46, 48, 54) with a total of 14 sub-studies explored the relationship between HbA1c-SD and cardiovascular disease mortality. There was heterogeneity among the studies (I²=78%, P<0.00001), so a random-effects model was used for analysis. The meta-analysis results showed that compared with type 2 diabetes patients with lower HbA1c-SD, the cardiovascular disease mortality of type 2 diabetes patients with higher HbA1c-SD increased by 27% (HR = 1.27, 95%CI 1.17-1.37, P<0.00001), as shown in **Figure 6A**.

#### 3.3.2 HbA1c-CV and cardiovascular disease mortality

Four studies (31, 36, 51, 54) with a total of 9 sub-studies explored the relationship between HbA1c-CV and cardiovascular disease mortality. There was heterogeneity among the studies (I²=90%, P<0.00001), so a random-effects model was used for analysis. The meta-analysis results showed that compared with type 2 diabetes patients with lower HbA1c-CV, the cardiovascular disease mortality of type 2 diabetes patients with higher HbA1c-CV increased by 35% (HR = 1.35, 95%CI 1.16-1.57, P<0.00001), as shown in **Figure 6B**.

#### 3.3.3 HbA1c-HVS and cardiovascular disease mortality

One study (8) with a total of 3 sub-studies explored the relationship between HbA1c-HVS and cardiovascular disease mortality. There was heterogeneity among the studies (I²=94%, P<0.00001), so a random-effects model was used for analysis. The meta-analysis results showed that HbA1c-HVS had no effect on cardiovascular disease mortality in patients with type 2 diabetes (HR = 1.00, 95%CI 0.76-1.31, P<0.00001), as shown in **Figure 6C**.

### 3.4 Subgroup analyses

Subgroup analyses consistently showed a positive association between HbA1c variability and CVD risk, but the robustness of the indices differed. As presented in **Appendix Table S1**, the pooled effects for SD-HR and CV-HR were highly consistent (1.27 and 1.32, respectively) and remained directionally stable across all subgroups. In contrast, HVS-HR (1.31, 95% CI 0.97–1.78) and HGI-OR (1.47, 95% CI 0.98–2.20) did not reach conventional statistical significance; nevertheless, their effect directions were consistent, suggesting a potential association rather than a “true null”. Notably, in studies with < 1000 participants the ORs for both HVS and HGI were statistically significant, implying that insufficient statistical power may be the main reason for the lack of significance in the overall analysis. When stratified by sample size, the SD-HR was 1.16 (95%CI: 1.13-1.18) in studies with a sample size of ≥1000, compared with 1.31 (95%CI: 1.07-1.61) in studies with a sample size of <1000. Subgroup analysis by region revealed that the association was strongest in non-Chinese Asian regions (SD-HR=1.77, 95%CI: 1.49-2.10), followed by 1.15 (95%CI: 1.13-1.18) in China and 1.13 (95%CI: 1.09-1.17) in other countries. In terms of study design, the SD-HR was 1.38 (95%CI: 1.16-1.64) for prospective studies and 1.23 (95%CI: 1.11-1.35) for retrospective studies. Studies with a follow-up duration of ≥5 years showed a higher hazard ratio (SD-HR=1.10, 95%CI: 1.08-1.13). Additionally, subgroup analysis by glycemic variability quartiles indicated that the risk of CVD in the highest quartile (Q4) was significantly higher compared with the lowest quartile (Q1) (SD-HR=1.52, 95%CI: 1.03-2.25). In summary, the associations for SD and CV are the most robust, whereas the “non-significance” observed for HVS and HGI is more likely attributable to insufficient statistical power rather than to a genuine biological null effect.

Subgroup analyses stratified by region (China vs. Other Asian vs. Other countries), study design (prospective vs. retrospective) and sample size (<1000 vs. ≥1000) reduced I² from the overall 90%–93% to 26%, 0% and 36%–52%, respectively, indicating that geographic setting and design features are the main sources of heterogeneity. Similarly, for HVS and HGI, restricting to follow-up<5 years or to small-sample studies lowered I² to 0%–25%, showing that time span and statistical power are also key moderators. For example, the pooled CV-HR had I² = 93%, yet every subgroup showed a consistent direction (HR > 1), and the pooled HR fluctuated only between 1.29 and 1.35 after sequential exclusion, implying that heterogeneity reflects magnitude rather than direction. Full details are given in **Appendix A Table S1–S2**.

### 3.5 Sensitivity analysis

The results of the sensitivity analysis showed that the association between glycemic variability (SD, CV) and cardiovascular disease risk was generally robust. After excluding specific studies or changing the statistical model, there was no substantial change in the direction or significance of the pooled effect size. After excluding studies with short follow-up durations, the effect sizes of SD-HR and CV-HR changed slightly; however, their 95% confidence intervals (CIs) did not include 1, still maintaining statistical significance, and the heterogeneity remained at a high level. When re-analyzed using a fixed-effects model, the pooled results of SD-HR and CV-HR were basically consistent with those of the random-effects model, which further confirmed the stability of the results.

Sensitivity analysis for different outcome indicators showed that after excluding studies reporting only specific endpoints such as heart failure with preserved ejection fraction (HFpEF) or ischemic heart disease (IHD), there was no significant deviation in the effect size. Similar patterns were observed in the analysis of HVS-HR and HGI-OR: excluding low-quality studies or studies of specific types did not alter the original conclusions. In summary, the sensitivity analysis supports the reliability of the conclusion regarding the association between glycemic variability and CVD risk. Details are shown in **Appendix A Table S3-S4**.

### 3.6 Publication bias

In this study, funnel plot analysis and Egger’s test were used to assess the presence of publication bias, and the trim-and-fill method was applied to correct for publication bias. The analysis showed that there was publication bias in the CV-HR for CVD incidence (Egger’s test, p=0.002). After imputing 2 potentially missing studies, the effect size decreased slightly from 0.280 to 0.246 (95%CI: 0.112-0.380). Although the effect was slightly weakened, it remained statistically significant. The p-value remained significant, and heterogeneity was not reported. These results indicate the presence of mild publication bias, which did not alter the statistical significance of the original conclusion. No significant publication bias was detected for other indicators. Details are shown in **Appendix A Table S5-S6**.

## 4 Discussion

Based on a systematic review and meta-analysis of 31 prospective/retrospective cohorts comprising >540000 patients with type 2 diabetes, we found that HbA1c variability (SD and CV) is independently associated with incident cardiovascular events and mortality, with pooled hazard ratios consistently between 1.27 and 1.35. In contrast, HVS and HGI showed the same directional trend but did not reach statistical significance, indicating that SD and CV are more robust predictors. SD/CV can be calculated easily without extra laboratory costs and can be integrated instantly into existing electronic medical-record systems to help identify “hidden high-risk” patients, whereas HVS/HGI require standardized algorithms and validation in larger samples before they can be considered as novel clinical predictors. Unlike most previous studies that primarily focused on mean HbA1c, this study provides the first population-level evidence that HbA1c fluctuations per se significantly increase cardiovascular risk even when average glycemic control has reached the target level, offering a new precision-glycaemia management target beyond conventional mean HbA1c.

Assessment of glucose homeostasis based solely on fasting blood glucose cannot capture post-prandial hyperglycemia, and short-term monitoring such as random blood glucose is insufficient to evaluate the progression risk of diabetic chronic complications. HbA1c, on the other hand, reflects overall exposure to both fasting and post-prandial glucose and therefore provides a more comprehensive picture of total glycemic control. Consequently, we selected HbA1c variability as the primary focus of this investigation.

Although methods for quantifying HbA1c variability are increasingly diverse, there is currently no unified “gold standard”. The most commonly used and simplest methods are calculating the SD and its derived CV. Numerous studies have reported a significant association between SD/CV and the deterioration of cardiovascular outcomes in patients with T2DM. A meta-analysis including 23 studies found that HbA1c variability, as assessed by SD or CV, was significantly associated with the risk of macrovascular complications (13). Another meta-analysis, which included 40 studies and 4,102,589 participants, showed that each increase in a variability indicator (HbA1c SD or CV) was associated with a 20%-26% increase in cardiovascular risk (55). Similar conclusions have also been drawn from multiple large-scale multicenter observational studies both domestically and internationally (52, 56). Consistent with previous studies, the present study also found that both SD and CV were positively correlated with the progression of cardiovascular disease.

HVS and HGI have also emerged as novel indicators, providing deeper insights into glucose metabolism. HVS is a specific indicator used to quantify long-term blood glucose fluctuations. Unlike the simple calculation of SD or CV, the calculation method of HVS is designed to reduce the impact of the number of measurements and measurement time intervals on results, and focuses more on evaluating fluctuations in HbA1c values that exceed specific thresholds. Hsu H et al. conducted a longitudinal cohort study, which retrospectively reviewed the incidence of MACE in patients with T2DM. The study concluded that early management of HbA1c using HVS is crucial for reducing the risk of adverse cardiovascular events in patients. HGI serves as an important individualized risk marker (57). Different from indicators such as SD, CV, and HVS that directly measure the magnitude of blood glucose fluctuations, HGI predicts the risk of diabetic cardiovascular complications from the perspective of an individual’s glycation susceptibility to blood glucose. Previous studies have shown that HGI is closely associated with various cardiovascular events. A large-scale multicenter cohort study, which included 9,791 participants, found that there was a U-shaped association between HGI values and 5-year MACE risk—both low and high HGI values were associated with an increased risk of MACE (58). Even when the average blood glucose level was similar, patients with high HGI still had a significantly increased risk of complications.

This is the first study to integrate three observational cohorts evaluating HVS and four cohorts addressing HGI. It was found that there was no significant association between increased HVS and the progression of cardiovascular events. Although multiple previous studies have suggested that glycemic variability is a potential predictor of cardiovascular risk in patients with T2DM, this study did not confirm this association. The possible reasons for this include the following aspects: First, in the included observational studies, confounding factors such as smoking status, hypoxic environment, and inflammatory levels were fully adjusted for. To a certain extent, this may have weakened the apparent association between HVS and cardiovascular outcomes (59). Second, there may be a U-shaped curvilinear relationship between HVS and cardiovascular risk—meaning the risk increases significantly only at extreme levels of variability, while a general increase does not independently elevate the risk of events (60). Furthermore, although HVS, as an indicator for measuring glycemic variability, has advantages such as being unaffected by the number of measurements and time intervals, and having strong clinical interpretability (it can reflect the frequency characteristics of HbA1c variability), its neglect of the magnitude of variability may limit its ability to predict long-term cardiovascular risk. Meanwhile, at the genetic level, Mendelian randomization analyses based on the HGI strategy also found no significant association between hemoglobin-related genetic variations and cardiovascular risk (61). This result further supports the aforementioned conclusion. The possible reasons for this include insufficient power of instrumental variables, failure of hemoglobin changes driven by genetic variations to capture specific biological processes related to disease pathways, or the presence of pleiotropic effects that interfere with causal inference. Overall, the null findings for HVS and HGI are more plausibly attributed to technical factors (few studies, heterogeneous definitions, population-specific effect patterns) than to a true absence of association. Future individual-patient-data meta-analyses with harmonized algorithms are warranted.

Meanwhile, this study estimated the OR of HbA1c variability using datasets, and a significant overall effect was observed. When evaluating HbA1c-related risks, there were obvious differences between the results presented by the OR and the HR. OR is used to measure the strength of the association between an intervention and an outcome. Although it can comprehensively reflect the overall effect, it often tends to overestimate the actual risk; in addition, this indicator has static characteristics and is difficult to capture information on the dynamic changes in event incidence over time (62). In contrast, HR focuses on the temporal differences in event occurrence and can more intuitively reflect the impact of an intervention on the timing of specific events. HR is usually estimated using the Cox proportional hazards model, which is suitable for analyzing the effect of covariates on the “time to first event,” thereby describing the dynamic process of risk changes over time and can be regarded as a measure of instantaneous risk intensity (63). It is particularly important to note that compared with HR, OR often shows an overestimation of risk, and a similar phenomenon was observed in this study—this was particularly evident in the correlation analysis between HbA1c SD/CV and cardiovascular outcomes in patients with T2DM.

Currently, there is no consensus on the optimal follow-up duration for HbA1c monitoring. This study innovatively explored the impact of different follow-up durations on cardiovascular incidence and mortality. Subgroup analysis in this study used a median follow-up period of 5 years as the cutoff, suggesting that 5 years may be a more effective follow-up indicator. In addition, SD, HGI, and HVS showed an upward trend in risk from the lowest quartile to the highest quartile, while CV did not exhibit a similar trend.

The key point is that each index captures a distinct glycemic signature: SD/CV reflects the amplitude of oscillations, HVS captures the frequency of clinically relevant jumps, while HGI quantifies individual glycation susceptibility. This meta-analysis shows that amplitude (SD/CV) carries the strongest and most consistent signal for CVD, an observation that aligns with *in-vitro* data demonstrating that larger glucose excursions generate more superoxide than chronic hyperglycemia of the same mean (64). For HVS, although the pooled estimate did not reach statistical significance, the upper confidence limit still extended to a 78% excess risk. Mechanistic studies indicate that every >0.5% HbA1c swing activates the NLRP3 inflammasome and leaves a persistent epigenetic footprint (H3K9me3)—the so-called “metabolic memory” (65). On the other hand, HGI may link to cardiovascular injury independently of ambient glucose: a high HGI signifies rapid hemoglobin glycation, paralleling band-3 protein glycation on the red-cell membrane, which reduces erythrocyte deformability and predisposes to micro-vascular sludging (66). Thus, HGI operates via a “blood-rheology” axis rather than the classic glucose-toxicity axis, explaining why its association with CVD remains positive yet weaker than that of SD/CV.

This study is the first meta-analysis to explore the association between multiple HbA1c variability indicators (SD, CV, HVS, HGI) and cardiovascular disease-related risk from multiple perspectives. A total of 31 cohort studies were included, with Newcastle-Ottawa Scale (NOS) quality scores ranging from 6 to 9. This indicates that the included studies have an overall high methodological quality, which enhances the credibility of the results. The results of extensive subgroup analyses and sensitivity analyses are consistent, further supporting the robustness of the main conclusions.

However, this study still has several limitations. First, there are differences in the detection frequency, time intervals, measurement equipment, and methods of HbA1c among the original studies, which may introduce heterogeneity. Second, some potential confounding factors have not been fully adjusted for, which may interfere with the estimation of effect sizes. Furthermore, this study aims to synthesize evidence from observational studies to address questions concerning HbA1c variability in real-world settings. The controlled environment of an RCT, including fixed follow-up schedules and strict intervention protocols, inherently influences the pattern of HbA1c variability, making it less generalizable to the fluctuations that occur in routine clinical practice. Therefore, while RCTs are superior for establishing causality, their applicability to our research objective is limited and *post-hoc* analyses of RCTs were excluded. Finally, this study focused on cardiovascular outcomes and did not involve other typical diabetic microvascular and macrovascular complications such as retinopathy, neuropathy, and kidney disease; therefore, caution should be exercised when extrapolating the conclusions.

In conclusion, this study indicates that HbA1c variability is positively correlated with the progression of incidence and mortality of cardiovascular disease-related events in patients with T2DM. Individualized treatment based on HbA1c variability may be a key component of precision medicine for T2DM.

## 5 Conclusion

This study confirmed through meta-analysis that there is a significant positive correlation between HbA1c variability and cardiovascular complications as well as all-cause mortality in patients with T2DM. This result suggests that HbA1c variability should be regarded as an important and independent predictive risk factor for T2DM patients. In particular, CV, SD, and HGI can serve as significant indicators for predicting the risk of CVD occurrence and mortality, and thus deserve greater attention in clinical practice and risk stratification.

## Data Availability

The original contributions presented in the study are included in the article/**Supplementary Material**. Further inquiries can be directed to the corresponding authors.
