# Supplementary material for: Glycemic variability of glycated hemoglobin in patients with type 2 diabetes mellitus and the risk of cardiovascular diseases: a latest systematic review and meta-analysis
Source: Front Endocrinol (Lausanne). 2025 Nov 6;16:1698360. doi: 10.3389/fendo.2025.1698360 (PMC12629938; doi:10.3389/fendo.2025.1698360)
Supplement: Supplementary file 1 [file DataSheet1.docx]

**Appendix A**

**Table 1**  Subgroup analyses for SD、CV HGI and HVS for CVD Incidence Rate.

| Subgroup | Studies | Pooled HR/OR (95% CI) | *I^2^* (*p* value) |
| --- | --- | --- | --- |
| ***SD-HR*** | | | |
| **Index** | | | |
| Adjusted SD | Ma C et al.(2022)/ | 1.25（1.14-1.38） | *I^2^* = 0%（*P* = 0.41） |
| SD | Bonke FC et al.(2016)/  Bouchi R et al.(2012) /  Cardoso CRL et al.(2018)/  Gu J et al.(2018)/  Lee MY et al.(2017)//  Luk AO et al.(2013) | 1.28（1.17-1.40） | *I^2^* = 91%（*P* < 0.01） |
| **CVD Outcome** | | | |
| incidence of HFpEF | Gu J et al.(2018) | 1.75（1.00-3.07） | / |
| incidence of CVD | Bonke FC et al.(2016)/  Bouchi R et al.(2012) /  Cardoso CRL et al.(2018)/  Lee MY et al.(2017)/  Luk AO et al.(2013)/  Ma C et al.(2022)/  Maajani K et al.(2025)/  Manosroi W et al.(2023)/  Shen Y et al.(2021)/  Teh XR et al.(2025)/  Wan EY et al.(2016)/  Wan EYF et al.(2020) | 1.27（1.17-1.38） | *I^2^* = 90%（*P* < 0.01） |
| **Sample Size** | | | |
| ＜1000 | Bouchi R et al.(2012)/  Bouchi R et al.(2012)/  Cardoso CRL et al.(2018)/  Gu J et al.(2018) | 1.31（1.07-1.61） | *I^2^* = 36%（*P* = 0.17） |
| ≧1000 | Bonke FC et al.(2016)/  Lee MY et al.(2017)/  Luk AO et al.(2013)/  Ma C et al.(2022)/  Maajani K et al.(2025)/  Manosroi W et al.(2023)/  Shen Y et al.(2021)/  Teh XR et al.(2025)/  Wan EY et al.(2016)/  Wan EYF et al.(2020) | 1.16 (1.13, 1.18） | *I^2^* = 92%（*P* < 0.01） |
| **Area** | | | |
| **China** | Gu J et al.(2018)/  Lee MY et al.(2017)/  Luk AO et al.(2013)/  Ma C et al.(2022)/  Wan EY et al.(2016)/  Wan EYF et al.(2020) | 1.15 (1.13, 1.18） | *I^2^* = 26%（*P* = 0.22） |
| **Other Asian** | Bouchi R et al.(2012)/  Manosroi W et al.(2023)/  Teh XR et al.(2025) | 1.77 (1.49, 2.10） | *I^2^* = 15%（*P* = 0.32） |
| **Other countries** | Bonke FC et al.(2016)/  Maajani K et al.(2025)/  Shen Y et al.(2021)/  Cardoso CRL et al.(2018) | 1.13 (1.09, 1.17） | *I^2^* = 96%（*P<* 0.01） |
| **Other countries(TIME)** |  |  |  |
| median of 5 years | Bonke FC et al.(2016)/  Cardoso CRL et al.(2018) | 0.90 (0.85, 0.94） | *I^2^* = 74%（*P* = 0.02） |
| ≧median of 5 years | Maajani K et al.(2025)/  Shen Y et al.(2021)/ | 1.38 (1.31, 1.45） | *I^2^* = 64%（*P* = 0.03） |
| **Design** |  |  |  |
| prospective | Cardoso CRL et al.(2018)/  Ma C et al.(2022)/  Luk AO et al.(2013)/  Manosroi W et al.(2023)  Wan EYF et al.(2020) | 1.38（1.16-1.64） | *I^2^* = 71%（*P* < 0.01） |
| retrospective | Bonke FC et al.(2016)/  Bouchi R et al.(2012)/  Gu J et al.(2018)/  Maajani K et al.(2025)/  Shen Y et al.(2021)/  Teh XR et al.(2025)/  Wan EY et al.(2016) | 1.23（1.11-1.35） | *I^2^* = 92%（*P* < 0.01） |
| **Time** | | | |
| median of 5 years | Bouchi R et al.(2012)/  Maajani K et al.(2025)/  Manosroi W et al.(2023)/  Shen Y et al.(2021) | 1.45 (1.38, 1.51） | *I^2^* = 70%（*P <* 0.01） |
| ≧median of 5 years | Bonke FC et al.(2016)/  Cardoso CRL et al.(2018)/  Gu J et al.(2018)/  Lee MY et al.(2017)/  Luk AO et al.(2013)/  Teh XR et al.(2025)/  Wan EYF et al.(2020) | 1.10 (1.08, 1.13） | *I^2^* = 90%（*P* < 0.01） |
| **Quartile** | | | |
| Q2/Q1 | Bonke FC et al.(2016) substudy1/  Bouchi R et al.(2012) substudy1/  Lee MY et al.(2017) substudy1/  Ma C et al.(2022) substudy1/  Manosroi W et al.(2023) substudy1/  Shen Y et al.(2021) substudy2 | 1.23（0.91-1.65） | *I^2^* = 93%（*P* < 0.01） |
| Q3/Q1 | Bonke FC et al.(2016) substudy2/  Bouchi R et al.(2012) substudy2/  Lee MY et al.(2017) substudy2/  Ma C et al.(2022) substudy2/  Manosroi W et al.(2023) substudy2/  Shen Y et al.(2021) substudy3 | 1.28（0.85-1.93） | *I^2^* =94%（*P* < 0.01） |
| Q4/Q1 | Bouchi R et al.(2012) substudy3/  Ma C et al.(2022) substudy3/  Manosroi W et al.(2023) substudy3/  Shen Y et al.(2021) substudy4 | 1.52（1.03-2.25） | *I^2^* =76%（*P* < 0.01） |
| ***HVS-HR*** | | | |
| **Sample Size** | | | |
| ＜1000 | Zhang F et al.(2023) | 1.64（1.25-2.17） | *I^2^* = 59%（*P* = 0.12） |
| ≧1000 | Kim H et al.(2023)/  Li S et al.(2020)-1 | 1.09 (0.93, 1.28） | *I^2^* = 84%（*P* < 0.01） |
| **Area** | | | |
| Other Country | Li S et al.(2020)-1 | 2.38（1.61-3.52） | */* |
| Non-Asia | Kim H et al.(2023) | 0.93（0.78-1.11） | *I^2^* = 0%（*P* = 0.93） |
| China | Zhang F et al.(2023) | 1.64（1.25-2.17） | *I^2^* = 59%（*P* = 0.12） |
| **Time** | | | |
| <median of 5 years | Zhang F et al.(2023) | 1.64（1.25-2.17） | *I^2^* = 59%（*P* = 0.12） |
| ≧median of 5 years | Li S et al.(2020)-1/  Kim H et al.(2023) | 1.09 (0.93, 1.28） | *I^2^* = 84%（*P* < 0.01） |
| ***HGI-OR*** |  |  |  |
| **Time** |  |  |  |
| < median of 5 years | Ahn CH et al.(2017) | 2.21（1.48-3.30） | *I^2^* = 25%（*P* = 0.25） |
| ≧median of 5 years | Kim MK et al.(2018) | 1.15（0.77-1.72） | *I^2^* =54%（*P* = 0.11） |
| **Quartile** |  |  |  |
| Q2/Q1 | Ahn CH et al.(2017) substudy1/  Kim MK et al.(2018) substudy1 | 1.28（0.72-2.27） | *I^2^* = 62%（*P* = 0.11） |
| Q3/Q1 | Ahn CH et al.(2017) substudy2/  Kim MK et al.(2018) substudy2 | 1.58（0.52-4.83） | *I^2^* =89%（*P <* 0.01） |
| Q4/Q1 | Kim MK et al.(2018) substudy3 | 1.74（1.08-2.80） | */* |
| ***CV-HR*** | | | |
| **CVD Outcome** | | | |
| incidence of HFpEF | Gu J et al.(2018) | 1.60（1.06-2.42） | */* |
| incidence of CVD | Bouchi R et al.(2012)/  Cardoso CRL et al.(2018)/  Ma C et al.(2022)/  Moosaie F et al.(2021)/  Shen Y et al.(2021)/  Teh XR et al.(2025)/  Wan EY et al.(2016) | 1.31（1.16-1.48） | *I^2^* = 93%（*P* < 0.01） |
| **Sample Size** | | | |
| ＜1000 | Bouchi R et al.(2012)/  Cardoso CRL et al.(2018)/  Gu J et al.(2018) | 1.32（1.06-1.65） | *I^2^* = 50%（*P* = 0.07） |
| ≧1000 | Ma C et al.(2022)/  Moosaie F et al.(2021)/  Shen Y et al.(2021)/  Teh XR et al.(2025)/  Wan EY et al.(2016) | 1.02 (1.01, 1.02） | *I^2^* = 95%（*P* < 0.01） |
| **Design** | | | |
| prospective | Cardoso CRL et al.(2018)/  Ma C et al.(2022) | 1.11（0.99-1.24） | *I^2^* = 0%（*P =* 0.53） |
| retrospective | Bouchi R et al.(2012)/  Shen Y et al.(2021)/  Teh XR et al.(2025)/  Wan EY et al.(2016) | 1.47（1.25-1.73） | *I^2^* = 96%（*P* < 0.01） |
| Case-cohort study | Moosaie F et al.(2021) | 1.33（1.14-1.55） | *I^2^* = 0%（*P* = 0.45） |
| **Time** | | | |
| <median of 5 years | Bouchi R et al.(2012)/  Shen Y et al.(2021) | 1.49（1.25-1.78） | *I^2^* = 88%（*P* < 0.01） |
| ≧median of 5 years | Gu J et al.(2018)/  Wan EY et al.(2016)/  Cardoso CRL et al.(2018)/  Moosaie F et al.(2021)/  Teh XR et al.(2025) | 1.01 (1.00, 1.02） | *I^2^* =79%（*P* < 0.01） |
| **Area** |  |  |  |
| Other countries | Cardoso CRL et al.(2018)/  Moosaie F et al.(2021)/  Shen Y et al.(2021) | 1.28 (1.24, 1.32） | *I^2^* = 82%（*P* < 0.01） |
| Other Asian | Bouchi R et al.(2012)/  Teh XR et al.(2025) | 3.69（2.19-6.22） | *I^2^* = 0%（*P* = 0.87） |
| China | Gu J et al.(2018)/  Ma C et al.(2022)/  Wan EY et al.(2016) | 1.06（0.84-1.33） | *I^2^* = 40%（*P* = 0.15） |
| **Other countries(Design)** |  |  |  |
| prospective | Cardoso CRL et al.(2018) | 1.13 (1.01, 1.28） | *I^2^* = 0%（*P* = 0.61） |
| retrospective | Shen Y et al.(2021)substudy1/  Shen Y et al.(2021)substudy4 | 1.21 (1.17, 1.26） | *I^2^* = 59%（*P* = 0.12） |
| Case-cohort study | Moosaie F et al.(2021) | 1.33 (1.14, 1.55） | *I^2^* = 0%（*P* = 0.45） |
| **Quartile** | | | |
| Q2/Q1 | Bouchi R et al.(2012) substudy1/  Ma C et al.(2022) substudy1/  Moosaie F et al.(2021) substudy2/  Shen Y et al.(2021) substudy2 | 1.33（1.01-1.74） | *I^2^* = 56%（*P* = 0.08） |
| Q3/Q1 | Bouchi R et al.(2012) substudy2/  Ma C et al.(2022) substudy2/  Moosaie F et al.(2021) substudy3/  Shen Y et al.(2021) substudy3 | 1.44（1.03-2.02） | *I^2^* =71%（*P =* 0.02） |
| Q4/Q1 | Bouchi R et al.(2012) substudy3/  Ma C et al.(2022) substudy3/  Moosaie F et al.(2021) substudy4/  Shen Y et al.(2021) substudy4 | 1.30（1.03-1.65） | *I^2^* =52%（*P* = 0.10） |
| ***HVS-HR*** |  |  |  |
| **Sample Size** |  |  |  |
| ＜1000 | Zhang F et al.(2023) | 1.64（1.25-2.17） | *I^2^* = 59%（*P* = 0.12） |
| ≧1000 | Li S et al.(2020)-1/  Kim H et al.(2023) | 1.09 (0.93, 1.28） | *I^2^* = 84%（*P* < 0.01） |
| **Area** |  |  |  |
| Other Country | Li S et al.(2020)-1 | 2.38（1.61-3.52） | */* |
| Non-Asia | Kim H et al.(2023) | 0.93（0.78-1.11） | *I^2^* = 0%（*P* = 0.93） |
| China | Zhang F et al.(2023) | 1.64（1.25-2.17） | *I^2^* = 59%（*P* = 0.12） |
| **Time** |  |  |  |
| <median of 8 years | Li S et al.(2020)-1/  Zhang F et al.(2023) | 1.73 (1.48, 2.04） | *I^2^* = 63%（*P* = 0.07） |
| ≧median of 8 years | Kim H et al.(2023) | 0.93 (0.78, 1.11） | *I^2^* =0%（*P* = 0.93） |
| ***HGI-OR*** |  |  |  |
| **Time** |  |  |  |
| median of 5 years | Zhang F et al.(2023) | 2.21（1.48-3.30） | *I^2^* = 25%（*P* = 0.25） |
| ≧median of 5 years | Kim H et al.(2023)/  Li S et al.(2020)-1 | 1.15（0.77-1.72） | *I^2^* =54%（*P* = 0.11） |
| **Quartile** |  |  |  |
| Q2/Q1 | Li S et al.(2020)-1 | 1.28（0.72-2.27） | *I^2^* = 62%（*P* = 0.11） |
| Q3/Q1 | Kim H et al.(2023) | 1.58（0.52-4.83） | *I^2^* =89%（*P <* 0.01） |
| Q4/Q1 | Zhang F et al.(2023) | 1.74（1.08-2.80） | */* |

SD: standard deviation; CV: coefficient of variation; HVS: HbA1c variability score; HGI: Hemoglobin glycation index; HR: hazard ratio; OR: Odds Ratio.

**Table 2**  Subgroup analyses for SD and CV for CVD Mortality Rate.

| Subgroup | Studies | Pooled HR/OR (95% CI) | *I^2^* (*p* value) |
| --- | --- | --- | --- |
| ***SD-HR*** | | | |
| **Sample Size** | | | |
| ＜1000 | Cardoso CRL et al.(2018)/  Takao T et al.(2014) | 2.16（1.32-3.55） | *I^2^* = 81%（*P* < 0.01） |
| 1000-10000 | Kaze AD et al.(2020)/  Wu TE et al.(2022) | 1.14（0.96-1.35） | *I^2^* = 8%（*P* = 0.35） |
| ≧10000 | Ceriello A et al.(2022)/  Wan EY et al.(2016)/  Wan EYF et al.(2020) | 1.26（1.16-1.37） | *I^2^* = 88%（*P* < 0.01） |
| **Area** | | | |
| Other countries | Cardoso CRL et al.(2018)/  Ceriello A et al.(2022)/  Kaze AD et al.(2020) | 1.20（1.15-1.25） | *I^2^* = 42%（*P* = 0.10） |
| Other Asian countries | Takao T et al.(2014) | 4.49（2.56-7.87） | *I^2^* = 26%（*P* = 0.26） |
| China | Wu TE et al.(2022)/  Wan EY et al.(2016)/  Wan EYF et al.(2020) | 1.36（1.32-1.41） | *I^2^* = 38%（*P* = 0.20） |
| **Design** |  |  |  |
| prospective | Cardoso CRL et al.(2018)/  Kaze AD et al.(2020)/  Wu TE et al.(2022)/  Wan EYF et al.(2020) | 1.36（1.32-1.41） | *I^2^* = 35%（*P* = 0.16） |
| retrospective | Ceriello A et al.(2022)/  Wan EY et al.(2016)/  Takao T et al.(2014) | 1.30（1.16-1.46） | *I^2^* = 83%（*P* < 0.01） |
| **Time** | | | |
| median of 5 years | Ceriello A et al.(2022) | 1.21（1.11-1.33） | *I^2^* = 79%（*P* < 0.01） |
| median of 5-9 years | Kaze AD et al.(2020)/  Wan EY et al.(2016)/  Wan EYF et al.(2020) | 1.26（1.15-1.39） | *I^2^* =62%（*P* = 0.03） |
| ≧median of 9 years | Cardoso CRL et al.(2018)/  Takao T et al.(2014)/  Wu TE et al.(2022) | 1.84（1.28-2.65） | *I^2^* =76%（*P* < 0.01） |
| **Quartile** | | | |
| Q2/Q1 | Ceriello A et al.(2022) substudy1/  Kaze AD et al.(2020) substudy1/  Takao T et al.(2014) substudy1 | 1.12（1.05-1.19） | *I^2^* =33%（*P* = 0.22） |
| Q3/Q1 | Ceriello A et al.(2022) substudy2/  Kaze AD et al.(2020) substudy2/  Takao T et al.(2014) substudy2 | 1.49（0.91-2.43） | *I^2^* =83%（*P* < 0.01） |
| Q4/Q1 | Ceriello A et al.(2022) substudy3/  Kaze AD et al.(2020) substudy3/  Takao T et al.(2014) substudy3 | 1.57（0.92-2.69） | *I^2^* =82%（*P* < 0.01） |
| ***CV-HR*** | | | |
| **Sample Size** | | | |
| ＜1000 | Takao T et al.(2014)/  Cardoso CRL et al.(2018) | 1.30（1.08-1.57） | *I^2^* = 65%（*P* = 0.02） |
| ≧1000 | Wan EY et al.(2016)/  Lin CC et al.(2024) | 1.02（1.02-1.03） | *I^2^* = 84%（*P* < 0.01） |
| **Design** | | | |
| prospective | Cardoso CRL et al.(2018) | 1.24（1.07-1.43） | *I^2^* = 0%（*P =* 0.96） |
| retrospective | Takao T et al.(2014)/  Lin CC et al.(2024)/  Wan EY et al.(2016) | 1.40（1.17-1.69） | *I^2^* = 92%（*P* < 0.01） |
| **Time** | | | |
| median of 5 years | Lin CC et al.(2024) | 1.58（1.27-1.96） | *I^2^* = 61%（*P* = 0.08） |
| ≧median of 5 years | Cardoso CRL et al.(2018)/  Wan EY et al.(2016)/  Takao T et al.(2014) | 1.02 (1.02, 1.03） | *I^2^* =84%（*P* < 0.01） |
| ≧median of 5 years(Quartile) | | | |
| High vs. Low SD | Cardoso CRL et al.(2018)/  Wan EY et al.(2016) | 1.02 (1.02, 1.03） | *I^2^* = 69%（*P* = 0.04） |
| Others | Takao T et al.(2014) | 3.31 (1.73, 6.33） | *I^2^* =0%（*P =* 0.33） |
| **Area** |  |  |  |
| Other country | Cardoso CRL et al.(2018)/  Takao T et al.(2014) | 1.30（1.08-1.57） | *I^2^* = 65%（*P* = 0.02） |
| HongKong, China | Wan EY et al.(2016)/  Lin CC et al.(2024) | 1.02（1.02-1.03） | *I^2^* = 84%（*P* < 0.01） |

SD: standard deviation; CV: coefficient of variation; HR: hazard ratio; OR: Odds Ratio.

**Table 3.** Sensitivity analysis of the association between HbA1c variability and Incidence of CVD

| Type analysis | Trial | Pooled HR/OR (95% CI) | *I^2^* (*p* value) |
| --- | --- | --- | --- |
| ***SD-HR*** | | | |
| Excluding trials | Bonke FC et al.(2016)/  Shen Y et al.(2021) | 1.17 (1.14, 1.20） | *I^2^* = 69%（*P* < 0.01） |
| Excluding trials with follow-up ＜5 years | Bouchi R et al.(2012)/  Maajani K et al.(2025)/  Manosroi W et al.(2023)/  Shen Y et al.(2021) | 1.16（1.14-1.18）/  1.15（1.13-1.17）/  1.15（1.13-1.17）/  1.12（1.10-1.14） | *I^2^* = 91%（*P* < 0.01）/  *I^2^* = 90%（*P* < 0.01）/  *I^2^* = 90%（*P* < 0.01）/  *I^2^* = 87%（*P* < 0.01） |
| Excluding trials which reported RRs | Maajani K et al.(2025) | 1.15（1.13-1.17） | *I^2^* = 90%（*P* < 0.01） |
| Fixed model analysis | Cardoso CRL et al.(2018)/  Gu J et al.(2018)/  Luk AO et al.(2013)/  Maajani K et al.(2025)/  Shen Y et al.(2021)substudy1/T  eh XR et al.(2025)/  Wan EY et al.(2016)/  Wan EYF et al.(2020) | 1.16（1.14-1.18）/  1.16（1.14-1.18）/  1.15（1.13-1.18）  1.16（1.14-1.18）/  1.16（1.14-1.18）/  1.16（1.14-1.18）/  1.16（1.14-1.18）/  1.17（1.13-1.20） | *I^2^* = 91%（*P* < 0.01）/  *I^2^* = 90%（*P* < 0.01）/  *I^2^* = 90%（*P* < 0.01）/  *I^2^* = 91%（*P* < 0.01）/  *I^2^* = 90%（*P* < 0.01）/  *I^2^* = 90%（*P* < 0.01）/  *I^2^* = 90%（*P* < 0.01）/  *I^2^* = 90%（*P* < 0.01） |
| ***SD-OR*** |  |  |  |
| Excluding trials with low quality | / | / | / |
| Excluding trials with follow-up ＜10 years | Yang HK et al.(2015) | 1.31（0.78-2.22） | *I^2^* = 63%（*P* = 0.10） |
| Excluding trials which reported RRs | / | / | / |
| Excluding trials which reported IHD | Akselrod D et al.(2021) | 1.33（1.09-1.63） | *I^2^* = 37%（*P* = 0.20） |
| Excluding trials which reported CACS | Yang HK et al.(2015) | 1.31（0.78-2.22） | *I^2^* = 63%（*P* = 0.10） |
| ***CV-HR*** | | | |
| Excluding trials with low quality | / | / | / |
| Excluding trials with follow-up ＜5 years | Wan EY et al.(2016)/  Shen Y et al.(2021)/  Bouchi R et al.(2012) | 1.22 (1.12, 1.33） | *I^2^* = 56%（*P* = 0.01） |
| Excluding trials which reported RRs | / | / | */* |
| Excluding trials which reported HFpEF | Gu J et al.(2018) | 1.02（1.01-1.03） | *I^2^* = 93%（*P* ＜ 0.01） |
| Fixed model analysis | Cardoso CRL et al.(2018)/  Gu J et al.(2018)/  Moosaie F et al.(2021) substudy1/  Shen Y et al.(2021) substudy1/  Teh XR et al.(2025)/  Wan EY et al.(2016) | 1.02（1.01-1.03）/  1.02（1.01-1.03）/  1.02（1.01-1.03）/  1.02（1.01-1.02）/  1.02（1.01-1.03）/  1.28（1.23-1.32） | *I^2^* = 94%（*P* ＜ 0.01）/  *I^2^* = 93%（*P* ＜ 0.01）/  *I^2^* = 93%（*P* ＜ 0.01）/  *I^2^* = 93%（*P* ＜ 0.01）/  *I^2^* = 93%（*P* ＜ 0.01）/  *I^2^* = 77%（*P* ＜ 0.01） |
| ***CV-OR*** |  |  |  |
| Excluding trials with low quality | / | / | / |
| Excluding trials with follow-up ＜5 years | Li S et al.(2020)-2/  Liu X et al.(2024)/  Sato M et al.(2021) | 1.41（1.18-1.68）/  1.41（1.21-1.65） | *I^2^* = 10%（*P* = 0.35）/  *I^2^* = 0%（*P* = 0.46） |
| Excluding trials which reported RRs | / | / | */* |
| Fixed model analysis | Li S et al.(2020)-2/  Liu X et al.(2024)/  Yang HK et al.(2015) | 1.47（1.17-1.84）/  1.38（1.21-1.57） | *I^2^* = 21%（*P* = 0.28）/  *I^2^* = 0%（*P* = 0.44） |
| ***HVS-HR*** |  |  |  |
| Excluding trials with | Li S et al.(2020)-1/  Zhang F et al.(2023) | 0.93 (0.78, 1.11） | *I^2^* = 0%（*P=* 0.93） |
| Excluding trials with follow-up ＜5 years | Zhang F et al.(2023) | 1.09（0.93-1.28） | *I^2^* = 84%（*P* ＜ 0.01） |
| Excluding trials which reported RRs | / | / | */* |
| Fixed model analysis | Li S et al.(2020)-1/  Zhang F et al.(2023) | 1.23（1.08-1.39）/  1.09（0.93-1.28） | *I^2^* = 82%（*P* ＜ 0.01）/  *I^2^* = 84%（*P* ＜ 0.01） |
| ***HGI-OR*** |  |  |  |
| Excluding trials with low quality | / | / | / |
| Excluding trials with follow-up ＜5 years | Ahn CH et al.(2017) | 1.15（0.88-1.51） | *I^2^* = 54%（*P* = 0.11） |
| Excluding trials which reported RRs | / | / | */* |
| Fixed model analysis | / | / | */* |

SD: standard deviation; CV: coefficient of variation; HVS: HbA1c variability score; HGI: Hemoglobin glycation index; HR: hazard ratio; RR: relative ratio; OR: Odds Ratio.

**Table 4.** Sensitivity analysis of the association between HbA1c variability and Mortality of CVD.

| Type analysis | Trial | Pooled HR/OR (95% CI) | *I^2^* (*p* value) |
| --- | --- | --- | --- |
| ***SD-HR*** | | | |
| Excluding trials with low quality | / | / | / |
| Excluding trials with follow-up ＜5 years | Ceriello A et al.(2022) | 1.35（1.31-1.40） | *I^2^* = 69%（*P* < 0.01） |
| Excluding trials which reported RRs | / | / | / |
| Fixed model analysis | Cardoso CRL et al.(2018)/  Wan EY et al.(2016)/  Wan EYF et al.(2020)/  Wu TE et al.(2022) | 1.30（1.26-1.33）/  1.30（1.26-1.33）/  1.22（1.18-1.27）/  1.29（1.26-1.33） | *I^2^* = 82%（*P* < 0.01）/  *I^2^* = 80%（*P* < 0.01）/  *I^2^* = 69%（*P* < 0.01）/  *I^2^* = 80%（*P* < 0.01） |
| ***CV-HR*** | | | |
| Excluding trials with low quality | / | / | / |
| Excluding trials with follow-up ＜5 years |  | 1.22 (1.12, 1.33） | *I^2^* = 56%（*P* = 0.01） |
| Excluding trials which reported RRs | / | / | */* |
| Fixed model analysis | Cardoso CRL et al.(2018)/  Lin CC et al.(2024)/  Wan EY et al.(2016) | 1.02（1.02-1.03）/  1.02（1.02-1.03）/  1.26（1.19-1.34） | *I^2^* = 92%（*P* ＜ 0.01）/  *I^2^* = 84%（*P* ＜ 0.01）/  *I^2^* = 78%（*P* ＜ 0.01） |

SD: standard deviation; CV: coefficient of variation; HR: hazard ratio; RR: relative ratio; OR: Odds Ratio.

**Table 5:** Egger's Test for the Association Between Glycemic Variability and Adverse Cardiovascular Events in Patients with Type 2 Diabetes.

| Indicators | *P-*value |
| --- | --- |
| SD-HR for CVD Incidence | 0.075 |
| CV-HR for CVD Incidence | 0.002 |
| SD-HR for CVD Mortality | 0.751 |

**Table 6:** Interpretation of the Egger's Test for CV-HR for CVD Incidence.

| Metric | Before Trim & Fill | After Trim & Fill | Interpretation |
| --- | --- | --- | --- |
| Effect Size | 0.280(0.163,0.396) | 0.246(0.112,0.380) | Weakened but remains significant |
| P-value | <0.0001 | Still significant | Significance unchanged |
| Publication Bias | / | 2 studies imputed | Mild publication bias present |
